# A Conserved Enhancer Locus in Extrachromosomal DNA and Homogeneously Staining Regions Activates *MYC* Transcription in Group 3 Medulloblastoma

**DOI:** 10.1158/0008-5472.CAN-25-4691

**Published:** 2026-04-22

**Authors:** Jake D. Friske, Flore Cuisin, Paloma Guernalec, Hayden A. Malone, Stephanie Nance, Declan Bennett, Steven J. Burden, Ti-Cheng Chang, Hao Shi, Justin S. Williams, Virginia Valentine, Barbara d.S. Passaia, Bensheng Ju, Modupeore O. Adetunji, Paul Geeleher, Brian J. Abraham, Gang Wu, Chunliang Li, Martine F. Roussel

**Affiliations:** 1 Department of Tumor Cell Biology, St. Jude Children’s Research Hospital, Memphis, Tennessee.; 2 St. Jude Graduate School of Biomedical Sciences, Memphis, Tennessee.; 3 Magistère Européen de Génétique, Universite Paris Cite, Paris, France.; 4 Department of Oncology, St. Jude Children’s Research Hospital, Memphis, Tennessee.; 5 Department of Computational Biology, St. Jude Children’s Research Hospital, Memphis, Tennessee.; 6 Cytogenetics Core Facility, St. Jude Children’s Research Hospital, Memphis, Tennessee.; 7 Center for Applied Bioinformatics, St. Jude Children’s Research Hospital, Memphis, Tennessee.; 8 Department of Immunology, St. Jude Children’s Research Hospital, Memphis, Tennessee.

## Abstract

*MYC* is amplified on extrachromosomal DNA (ecDNA) or homogeneously staining regions (HSR) in group 3 medulloblastoma (G3-MB), conferring a poor prognosis. A better understanding of the mechanisms underlying *MYC* expression in ecDNA and HSRs could be leveraged to develop improved treatments for G3-MB. Using a structure–function approach, we identified and characterized an enhancer (*ecMYC E1*) that drives *MYC* activation specifically in G3-MB with *MYC*-amplified ecDNA or HSRs. The *ecMYC E1* locus exhibited enhancer hallmarks exclusively in *MYC*-amplified G3-MB but not in other *MYC*-dependent cancer cell lines, including those with *MYC* amplification. Silencing of the *ecMYC E1* enhancer significantly reduced *MYC* transcription, which was compensated for by increases in ecDNA copy number. Neuronal differentiation 1 and bromodomain-containing protein 4 interacted with each other and bound to *ecMYC E1*, looping the enhancer to the *MYC* promoter. Together, these findings define a mechanism that regulates amplified *MYC* gene expression within ecDNA or HSRs specifically in G3-MB.

**Significance:**

Combined structural and functional approaches identified a conserved enhancer activating *MYC* transcription exclusively in *MYC*-amplified ecDNA- and HSR-positive group 3 medulloblastoma, providing a potential therapeutic target for suppressing *MYC*.

## Introduction

Pediatric cancer is one of the leading causes of childhood mortality globally, and medulloblastoma is the most common malignant brain tumor in children, with more than 400 cases diagnosed in the United States each year (1). Medulloblastoma is divided into four molecularly distinct subgroups: Wingless, Sonic Hedgehog (SHH), group 3 (G3), and group 4. Each medulloblastoma subgroup arises from distinct cells of origin within the developing cerebellum (2), and tumors from each subgroup carry different prognoses, with G3 medulloblastoma (G3-MB) exhibiting the worst clinical outcome (2). SHH-MB and G3-MB are characterized by overexpression and/or amplification of *MYCN* and *MYC*, respectively, with oncogene amplification conferring significantly poorer responses to conventional therapy (3). Gene amplification can sometimes be visualized cytogenetically as homogeneously staining regions (HSR) within a chromosome. Alternatively, gene amplification can occur within extrachromosomal DNA (ecDNA) as circular amplicons lacking centromeres, first observed in the formation of “double minutes” (DM) in 1965 (4, 5).

ecDNAs replicate during the DNA synthetic phase of the cell cycle, but they randomly segregate as episomes to daughter cells during mitosis (6). Each ecDNA unit is 500 kilobases to 3 megabases in length and harbors one or more genes, often including oncogenes (*MYC*, *MYCN*, *EGFR*, and *MDM2*) or drug resistance genes [dihydrofolate reductase (*DHFR*)], as well as regulatory DNA elements, such as enhancer regions and long noncoding RNAs (6). ecDNAs are generally formed through complex rearrangements rather than simple circularization of amplified chromosomal loci (7). ecDNAs or HSRs containing oncogenes are frequently found in tumor cells, in which they can drive tumor progression and, in turn, serve as prognostic markers for significantly poorer clinical outcomes in pan-cancer analyses (8). ecDNA or HSR amplicons can be found in 20% of all cancer types (9) and at a disproportionately higher frequency in pediatric brain tumors (5). Tumors containing episomal DMs exhibit high genetic diversity and accelerated evolution because the random segregation of ecDNAs into daughter cells enables rapid adaptation to external stressors, such as chemotherapy or targeted therapies (10). An equivalent plasticity in stress responses is not as readily observed in tumor cells, in which gene amplification occurs within intrachromosomal HSRs, highlighting the selective advantages conferred by episomal replication (8). In addition to oncogene amplification, ecDNAs are characterized by increased open chromatin and poised enhancers, which facilitate enhanced transcription and oncogenicity (6). Clusters of ecDNA (known as “hubs”) further enable intermolecular enhancer–gene interactions to promote oncogene overexpression (11).

G3-MBs are characterized by a block in differentiation and unabated proliferation of neuronal progenitor cells, driven by *MYC* overexpression or amplification in 18% of cases (5). The *MYC* family of proto-oncogene transcription factors activates the expression of proliferative genes and regulates many cellular processes, such as growth, metabolism, and apoptosis (12). Enforced *Myc* overexpression combined with *Trp53* loss in cerebellar granule neuron progenitors is sufficient to drive G3-MB in mice (13). Although these models proved helpful in pinpointing the downstream consequences of *MYC* activation and in facilitating the development of novel therapies, they do not recapitulate *MYC* regulation by ecDNA amplification. In contrast, although genetically engineered mouse tumor models driven by *MYC* amplification in ecDNA (14) were experimentally generated by circularization of the *MYC* genomic locus, they fail to reflect the complex rearrangements and novel regulatory landscape observed in patient tumors with ecDNA (15). Gain or overexpression of orthodenticle homeobox genes *OTX1*, *OTX2*, or both, and expression of each, are correlated with clinical and pathologic classifications in G3-MB (16). These transcription factors are involved in the developing nervous system and contribute to the maintenance of stemness in *MYC*-amplified medulloblastoma (17). Only *OTX2* was found to be amplified on ecDNA in G3-MB (15).

We have now taken advantage of patient-derived established cell lines and tumor organoids of G3-MB to investigate *MYC* regulation in ecDNA- and HSR-positive tumors. Combining structural investigation with functional perturbation, we identified an enhancer locus (designated as *ecMYC E1*) that activates *MYC* transcription exclusively in *MYC*-amplified G3-MB but not in other *MYC*-driven cancer cell lines. Silencing of *ecMYC E1* inhibited *MYC* mRNA expression, which was compensated for by an increase in ecDNA copy number, restoring *MYC* transcript levels. This highlights the enhanced capacity of ecDNA-containing tumors to overcome external stressors and develop resistance to therapy compared with HSR-containing tumors. Bromodomain-containing protein 4 (BRD4) and neuronal differentiation 1 (NeuroD1) colocalize at the *ecMYC E1* locus in *MYC*-amplified G3-MB to facilitate *MYC* transcription, and their displacement led to functional silencing of *ecMYC E1* with a concomitant decrease in *MYC* expression. Disruption of the complex via bromodomain inhibition suppressed *ecMYC E1* activity, which might be leveraged as a therapeutic option in G3-MBs with *MYC* amplification in ecDNA or HSRs.

## Materials and Methods

### Cell culture

The human G3-MB cell line D425 (RRID: CVCL_1275), (from Duke University; ref. 18), was cultured according to ATCC handling guidelines in ultra-low attachment T75 (Corning, #3814) as tumor spheres in NeuroCult NS-A Basal Medium Human (STEMCELL Technologies, #05750) supplemented with proliferation supplement (STEMCELL Technologies, #05753), 1× B-27 minus vitamin A (Thermo Fisher Scientific, #12587-010), 1× N2 (Thermo Fisher Scientific, #17502-048), 1% penicillin/streptomycin, 1% GlutaMax (Thermo Fisher Scientific, #35050-061), 0.015% bovine serum albumin (BSA; Sigma-Aldrich, #A8412), 0.004% heparin (STEMCELL Technologies, #07980), and 20 ng/mL each of human recombinant epidermal growth factor (EGF, Thermo Fisher Scientific, #AF-100-15-500UG) and human fibroblast growth factor (FGF2, Thermo Fisher Scientific, #100-18B-50UG). The human G3-MB cell line, D458 (ATCC, #CRL-3632; RRID: CVCL_1161; ref. 18), was cultured as adherent cells in 10-cm tissue culture dishes (Falcon, #353003) in Dulbecco’s Modified Eagle Medium (Gibco, #10313-021) supplemented with 10% FBS (Thermo Fisher Scientific, #A5256701), 1% L-glutamine (Thermo Fisher Scientific, #25030081), and 1% penicillin–streptomycin (Thermo Fisher Scientific, #15140122). D341 (from Duke University) was cultured as tumor spheres in Eagle’s Minimal Essential Media (ATCC, #30-2003) supplemented with 20% FBS (Thermo Fisher Scientific, #A5256701), 1% L-glutamine (Thermo Fisher Scientific, #25030081), and 1% penicillin–streptomycin (Thermo Fisher Scientific, #15140122). D283 (from Duke University) was cultured in Eagle’s Minimal Essential Media (ATCC, #30-2003) supplemented with 10% FBS (Thermo Fisher Scientific, #A5256701), 1% L-glutamine (Thermo Fisher Scientific, #25030081), and 1% penicillin–streptomycin (Thermo Fisher Scientific, #15140122). The human G3-MB tumor organoids SJMB016880 and SJMB049291 (19) were cultured in ultra-low attachment T75 (Corning, #3814) as tumor spheres in tumor stem media (TSM) full media (20). Written informed consent was obtained from patients or legal representatives. All studies were conducted in accordance with the US Common Rule and were approved by the St. Jude Children’s Research Hospital Institutional Review Board under the approval number NBTP01. All cells were kept at 37°C in 5% CO_2_ and tested for *Mycoplasma* at each passage and quarterly for short tandem repeat fingerprinting.

### Fluorescence *in situ* hybridization

Fluorescence *in situ* hybridization (FISH) was performed as previously described (21) using probes targeting *MYC* (RP11-237F24), *OTX2* (CH17-288G22), or chromosome 8 control (D8Z2). Detection of *MYC* amplification by FISH assay utilized purified *MYC* promoter BAC DNA (RP11-237F24 /8q24 /128,690,817-128,824,097 mb Hg19) or *OTX2* promoter BAC DNA (CH17-288G22) labeled with red-dUTP (SEEBRIGHT 580, Enzo Life Sciences) by nick translation. Chromosome 8 control DNA (D8Z2) was labeled with green-dUTP (SEEBRIGHT 496, Enzo Life Sciences). Probes were combined and hybridized as red/green pairs to metaphase and interphase nuclei in a solution containing 50% formamide, 10% dextran sulfate, and 2× SCC. After overnight hybridization, slides were washed, stained with DAPI, and analyzed. Two hundred cells per sample were scored for the presence or absence of *MYC* or *OTX2*. One hundred fifty metaphase cells were scored for each type of amplification (DM vs. HSR). For ecDNA counting analyses, cells were analyzed in each channel (FITC and mCherry) for regions of interest (ROI) after imaging, with ROI diameters set to 0.5 μm. Colocalization was calculated by measuring the distance between ROI midpoints in the X, Y, and Z planes, using a threshold of 1 μm. A total of 101 cells were analyzed across each of the adenovirus-associated virus integration site-1 (AAVS1) and sg102 cell lines.

### DNA/RNA extraction

Up to 2 × 10^6^ cells were pelleted by centrifugation at 500 × *g* for 5 minutes in 1.5 mL Eppendorf tubes. DNA and RNA were extracted using the AllPrep DNA/RNA mini kit (QIAGEN, #80204) according to the manufacturer’s protocol.

### ecDNA reconstruction—Episomizer

EcDNAs from D425 cells were reconstructed using the Episomizer pipeline, as previously described (22). Raw whole-genome sequencing (WGS) reads were quality-controlled and aligned to the human reference genome assembly GRCh38 using the Parabricks BWA-MEM algorithm (arXiv:1303.3997v1; RRID: SCR_022192). CONSERTING was used to detect copy-number amplification segments (23), from which those with two SDs above the mean were considered highly amplified regions. The structural variants surrounding each segment were identified from soft-clipped and discordant reads using CREST algorithms (RRID: SCR_005257; ref. 24). Small segments bridging the neighboring segments were determined from actual reads spanning the breakpoints. The paths and orientations of segments across all possible combinations of episomes were determined using Episomizer (22). The final structure of the episomes was visualized by Circos plots (RRID: SCR_011798; ref. 25).

### ecDNA reconstruction—AmpliconArchitect

EcDNA detection and structural reconstruction were performed using AmpliconSuite (version 1.3.5). WGS FASTQ reads underwent quality control and were aligned to the GRCh38 human reference genome using the Burrows-Wheeler Aligner (RRID: SCR_010910; BWA version 0.7.15; ref. 26) with default parameters. Following alignment, BAM files were sorted and indexed using SAMtools (version 1.19; RRID: SCR_002105; ref. 27), and duplicate reads were marked using Picard MarkDuplicates (version 1.1; RRID: SCR_006525). To identify and characterize ecDNAs, we ran AmpliconSuite (version 1.3.5; bioRxiv 2024.05.06.592768), which integrates copy-number segmentation data generated by CNVkit (RRID: SCR_021917) with discordant read-pair analysis. This computational framework enabled the detection of focal amplifications characteristic of circular ecDNA elements and facilitated comprehensive structural reconstruction of the amplicon architecture.

### ecMYC E1 and MYC promoter copy-number variant analysis

The copy-number variant (CNV) analysis of the St. Jude model and patient data was performed as previously published (21). CNVs from WGS were called applying CONSERTING (23) using tumor/normal pairs when available. Additional samples were included based on overlap between the *ecMYC E1* enhancer and *MYC* promoter regions and segment-based CNV data from the Pediatric Dependency Map Consortium (PedDep; ref. 28) 2Q 2025 release. *MYC* amplification was defined as *MYC* copy number ≥6.

### 10x single-cell RNA sequencing and analysis

D425 cells were dissociated into a single-cell suspension and fluorescence-activated cell sorted for live singlets before processing for the 10x Genomics Chromium Next GEM Single Cell 3′ GEM version 3.1 (10x Genomics, #1000121; RRID: SCR_023672) according to the manufacturer's protocol with a targeted recovery of 7,000 cells. Microfluidics chips were run on the 10x Genomics Chromium X machine. Final libraries were run on an Illumina NovaSeq 6000 (RRID: SCR_016387). For gene expression sequencing, the filtered count matrices were read into the R package Seurat (version 4.1; RRID: SCR_007322). Features detected in fewer than five cells were removed from the dataset. Cells with abnormally low gene numbers (<200) or high mitochondrial read percentages (≥20%; potentially dead or damaged cells) were removed. Cells with abnormally high unique molecular identifier (UMI) counts (≥40,000, potentially multiple cells in a single droplet) were also removed. After filtering, 3,704 cells were retained with an average of 3,880 genes per cell (UMI median: 15,097; range: 508–39,916). After quality control, libraries were normalized with the NormalizeData function (scale.factor = 1 × 10^6^) in the Seurat R package (RRID: SCR_007322). Gene expression on Uniform Manifold Approximation and Projection for Dimension Reduction was visualized by the FeaturePlot function. The Pearson correlation coefficient between *OTX2* and *MYC* was calculated by the FeatureScatter function.

### OTX2 and NeuroD1 siRNA knockdown

The transfection mix was prepared using RNAiMAX reagent (Invitrogen, #13778-150) according to the manufacturer’s instructions, with siRNAs for OTX2 (Fisher Scientific, #s9931 and #s9932), NeuroD1 (#s9457 and #s9458), or control (Thermo Fisher Scientific, # 4390843).

### BRD4 inhibition using SJ432

D425, D458, or SJMB016880 cells were plated in a six-well plate at a cell concentration of 0.5 × 10^6^ cells per well in 4 mL of growth media. Cells were then treated with DMSO or 100 nmol/L of the BD2-specific BRD4 inhibitor, SJ432 (29), for 1, 8, or 16 hours. At the conclusion of treatment, cells were harvested, washed, and pelleted, and DNA/RNA was extracted as described above. Reverse transcriptase quantitative polymerase chain reaction (RT-qPCR) was performed as described below.

### Immunoblotting

Protein lysates were prepared from cell pellets resuspended in RIPA buffer [50 mmol/L Tris (pH 7.4), 150 mmol/L NaCl, 0.1% SDS, 1% sodium deoxycholate, 1% Triton X-100, and 1 mmol/L EDTA] supplemented with protease inhibitors (Pierce, #A32955) in a volume of lysis buffer equivalent to the estimated pellet volume. Samples were placed on ice for 30 minutes, sonicated, and centrifuged at 11,000 rpm for 15 minutes at 4°C using a refrigerated centrifuge. Supernatants were transferred to new tubes and kept on ice. Protein concentration was determined using the Pierce Bicinchoninic Acid (BCA) Protein Assay Kit (Thermo Fisher Scientific, #23225). A 6% BSA stock solution was prepared and used to generate a standard curve with final concentrations of 2, 1, 0.5, 0.25, and 0.125 µg/µL, along with a blank control. For each sample, 2 µL of protein lysate was mixed with 23 µL of PBS. BCA working reagent was prepared by mixing 1 part of reagent B with 50 parts of reagent A, and 500 µL of this reagent was added to each sample and standard. Samples were incubated at 37°C for 30 minutes, and absorbance was measured at 562 nm using a GENESYS 150 spectrophotometer (Thermo Fisher Scientific, #850-300000) and standard cuvettes. A standard curve was generated to determine protein concentrations using linear regression. Extracted proteins were kept at 4°C for immediate use or at −80°C for later analyses. Protein lysates were diluted to a final concentration of 20 µg per lane in a final volume of 20 µL, containing 4 µL of 5× loading buffer (BioLegend, #426311). Samples were adjusted with lysis buffer when needed, heated at 95°C for 5 minutes, immediately cooled on ice, and briefly centrifuged before loading. Proteins were separated by SDS-PAGE on NuPAGE 4% to 12% Bis-Tris gels (Fisher Scientific, #NP0335) using 1× MES SDS running buffer (Thermo Fisher Scientific, #NP0002). A total of 20 µL of each protein sample and 8 µL of molecular weight ladder (LICORbio, #926-98000) were loaded per well. Electrophoresis was carried out at 100 V for 3 to 4 hours. Proteins were transferred onto polyvinylidene difluoride (PVDF) membranes using a wet transfer system in 1× transfer buffer (25 mmol/L Tris base, 192 mmol/L glycine, and 20% methanol). The gel and membrane sandwich was assembled with presoaked sponges and Whatman filter paper. Transfers were performed at 100 V for 1 hour at 4°C. PVDF membranes were blocked in 5% nonfat dry milk prepared in Tris-buffered saline with Tween 20 (TBS-T) buffer for 1 hour at room temperature with gentle agitation. Membranes were incubated overnight at 4°C with primary antibodies diluted in 5% milk in TBS-T (see Supplementary Table S1 for the list of primary antibodies used). After three washes in TBS-T, membranes were incubated for 1 hour at room temperature with secondary antibodies diluted in 5% milk in TBS-T (see Supplementary Table S1 for the list of secondary antibodies used). Following three additional washes, signal detection was performed using enhanced chemiluminescence reagents (Thermo Fisher Scientific, #32106) and imaged with the LICOR Odyssey imaging system (RRID: SCR_023765; LICORbio).

### Flow cytometry cell-cycle analysis

One million D425 cells were washed once with PBS and resuspended in 1 mL of propidium iodide (PI) solution [0.05 mg/mL PI; 0.1% (w/v) sodium citrate; and 0.1% (v/v) Triton X-100], vortexed to mix, and incubated for 30 minutes at room temperature in the dark. After incubation, samples were treated with 10 μL of 0.2 mg/mL RNase (Thermo Fisher Scientific, #EN0531) for 30 minutes at RT. Fluorescence was acquired on a BD Symphony A5-SE flow cytometer (RRID: SCR_022674; BD Biosciences) controlled by DIVA software (version 9.6; RRID: SCR_001456). Fluorescence intensity was analyzed using ModFit software (Verity Software House; RRID: SCR_016106).

### Generation of dCas9–KRAB stable cell lines

High-titer lentiviruses were generated using the Lenti-dCas9-KRAB-blasticidin plasmid (RRID: Addgene_89567). Cells were infected at a multiplicity of infection (MOI) of five infectious units per cell, supplemented with 8 µg/mL polybrene (Sigma-Aldrich, #TR-1003). Cells were infected every 3 hours for a total of three infections. After 72 hours, 30 µg/mL blasticidin (InvivoGen, #ant-bl-05) was added to the media. Cells were under blasticidin selection for 10 days.

### Single-guide RNA constructs

Constructs targeting the *MYC* promoter were cloned into the lentiguide-puro backbone (RRID: Addgene_52963). Single-guide RNA (sgRNA) sequences targeting the *ecMYC E1* locus (chr8:127,299,000–127,300,250) were generated using CRISPick (RRID: SCR_025148; ref. 30) and cloned into the LVA-sgRNA-ametrine backbone (31) using NEBuilder HiFi DNA Assembly Master Mix (New England Biolabs, #E2621L) according to the manufacturer’s instructions. All sgRNA sequences are listed in Supplementary Table S2.

### CRISPR inhibition sgRNA library

The library targeting the regulatory region surrounding the *MYC* locus consisted of 10,115 sgRNAs (Supplementary Table S3). Each sgRNA was cloned into the lentiguide-puro vector (RRID: Addgene_52963) by the St. Jude Center for Advanced Genome Editing (CAGE).

### CRISPR inhibition screen

D425 cells were dissociated and infected at an MOI of 0.7 in biological triplicate. Three days after infection, each replicate was puromycin-selected at 2 µg/mL for 72 hours. After 72 hours, half of each culture was taken for initial library representation. Cells were cultured for a total of 14 passages. DNA was extracted from the first and last time points, and sgRNA representation was quantified by next-generation sequencing. sgRNA dropout analysis was performed using a modified version of the Model-based Analysis of Genome-wide CRISPR–Cas9 Knockout (MAGeCK; RRID: SCR_025016) developed by the CAGE (32). Detailed screening results are included in Supplementary Table S4.

### Assay for Transposase-Accessible Chromatin using sequencing

We used a modified version of the Omni Assay for Transposase-Accessible Chromatin using sequencing (ATAC-seq) protocol (33). ATAC-seq read quality was assessed with FastQC (version 0.11.9; RRID: SCR_014583). The Illumina adapter (“CTGTCTCTTATACACATCT”) and low-quality bases were trimmed, and reads shorter than 60 bp were removed using Trim Galore (version 0.6.10; RRID: SCR_011847) and Cutadapt (version 3.7; RRID: SCR_011841). Reads were aligned to the human genome (GRCh38) with Bowtie 1 (version 1.2.3; RRID: SCR_005476) using ATAC-seq–optimized parameters (-allow-contain -chunkmbs = 256 -l = 76 -k = 2 -m = 2 -X = 600 -best). Postalignment filtering removed low-quality, mitochondrial, and duplicate reads, as well as reads overlapping ENCODE blacklist regions, using Samtools (version 1.9; RRID: SCR_002105) and Bedtools (version 2.30.0; RRID: SCR_006646). Quality metrics, including transcription start site enrichment, nucleosome profiling, fraction of reads in peaks, and library complexity, were calculated using the SEAseq (version 3.1) quality control modules. Within SEAseq, genome coverage tracks were generated from paired-end BAM alignments by converting mapped fragments to paired BEDPE format, quantifying fragment coverage in 50 bp bins, and converting the resulting graphs to bigwig format using the UCSC bedGraphToBigWig command-line program (RRID: SCR_007708).

### ecMYC E1 targeting

G3-MB cells were infected with lentiviruses containing sgRNAs targeting the *ecMYC E1* enhancer (Supplementary Table S2) at a MOI of 8, supplemented with 8 µg/mL polybrene (Sigma-Aldrich, #TR-1003) 3 times. Forty-eight hours after the final infection, cultures were sorted for the top 10% of ametrine-expressing cells. Half of the sorted cells were used as the initial time point (doubling time 0, DT0), whereas the other half was returned to tissue culture. Cells were passaged, and aliquots were taken at DT3, DT7, DT10, and DT13 for DNA/RNA extraction and at DT13 for paired DNA/RNA FISH analysis.

### RT-qPCR

RT-qPCR was performed as previously described (34). We used TaqMan assay ID Hs00153408_m1 for human *MYC* and TaqMan assay ID Hs02786624_g1 for the human *GAPDH* normalization control.

### DNA qPCR

DNA was extracted from cells as described above. qPCR was performed with an Applied Biosystems QuantStudio 3 Real-Time PCR system (Applied Biosystems; RRID: SCR_018712) using Fast SYBR Green reagents (Applied Biosystems, #4385612) and primers specific to the ROI (see Supplementary Table S5). Threshold cycle values from triplicate measurements were averaged and normalized to the GAPDH internal control gene. Relative expression was quantified by the 2^−ΔΔCT^ method (35). Statistical analyses were performed in GraphPad PRISM Software versioin 10.0 (RRID: SCR_002798). Statistical significance was determined by a Student *t* test as indicated by the figure legends.

### Chromatin immunoprecipitation followed by next-generation sequencing (chromatin immunoprecipitation sequencing)

All samples were collected in biological duplicates. For each sample/replicate, 10 million cells were fixed for 5 minutes at room temperature in 1% formaldehyde using the Covaris truChIP Chromatin Shearing Kit (Covaris, #520154). Nuclei were isolated according to the manufacturer’s protocol, and chromatin was sheared using a Covaris M220 ultrasonicator (RRID: SCR_027073) in a Covaris milliTUBE (Covaris, #520128) with the following parameters: duty factor = 10, 200 cycles/burst, 10 minutes, and 6°C. Sheared chromatin was centrifuged at 8000× g for 10 minutes, and clarified chromatin was moved into a fresh 1.5 mL Eppendorf tube. Chromatin was amended to a final concentration of 50 mmol/L Tris-HCl (pH 7.4), 100 mmol/L NaCl, 1 mmol/L EDTA, 1% NP-40, 0.1% SDS, and 0.5% sodium deoxycholate, with protease inhibitors. Ten micrograms of antibody (Supplementary Table S1) was added to each respective chromatin sample and incubated overnight at 4°C with gentle rotation. Spike-in chromatin (Active Motif, #53083; RRID: AB_2737370) and antibody (Active Motif, #61686; RRID: AB_2737370) were added to each reaction according to the manufacturer’s protocol. The next day, 60 µL of Protein G magnetic beads (Invitrogen, #10003D) were added to each reaction and incubated for at least 4 hours at 4°C with gentle rotation. Samples were placed on a magnetic stand, and unbound chromatin was removed. Beads were washed twice with wash buffer 1 [50 mmol/L Tris-HCl (pH 7.4), 1 mmol/L EDTA, 1% NP-40, 0.1% SDS, and 0.5% sodium deoxycholate, with protease inhibitors] and washed once with wash buffer 2 [20 mmol/L Tris-HCl (pH 7.4), 10 mmol/L MgCl_2_, and 0.2% Tween-20, with protease inhibitors]. Beads were resuspended in wash buffer 2, transferred to a new 1.5 mL Eppendorf tube, and placed on a magnetic stand to remove the wash buffer. DNA was eluted and de-cross-linked in elution and de-cross-linking solution (1× Tris-Ethylenediaminetetraacetic acid (TE); 1% SDS; 5% proteinase K; and 400 mmol/L NaCl) at 65°C for 4 hours with shaking. DNA was precipitated using phenol, chloroform, and isopropyl alcohol, and libraries were constructed using the NEBNext Ultra II Library Prep Kit (New England Biolabs, #E7645S) and NEBNext Multiplex Oligos for Illumina (New England Biolabs, #73335S) according to the manufacturer’s protocol. Single-end reads (1 × 50) were obtained from all samples, and adapter oligos were trimmed by trim_galore (version 0.4.4, “—gzip”; RRID: SCR_011847) and aligned to the human genome hg38 (Gencode GRCh38.p12) by BWA (version 0.7.12-r1039, default parameters; RRID: SCR_010910; ref. 26). Duplicate reads were marked with biobambam2 (version 2.0.87; RRID: SCR_003308; ref. 36). BAM files were converted into bigWig coverage tracks by deepTools (version 3.5.6, default parameters) (RRID: SCR_016366; ref. 37). We observed reasonable peaks on the Integrative Genomics Viewer (IGV version 2.19.1; RRID: SCR_011793; ref. 38). NeuroD1 occupancy after silencing was determined using bamCompare from deepTools (version 3.5.6; RRID: SCR_016366) using 50 base pair bins within the 2 kilobase region chr8:127,299,000–127,301,000. The log_2_ fold change of H3K27ac histone modification enrichment was compared between regions defined as EIE14: chr3:111,554,750–111,556,750; *ecMYC E1*: chr8:127,299,000–127,301,000; *MYC* enhancer: chr8:127,735,000–127,738,000. Enrichment was calculated as log_2_(H3K27ac/Input), where H3K27ac and Input represent the summed read signal mapped to a matching region per sample.

### Cut under target and release using nuclease followed by next-generation sequencing

Cut under target and release using nuclease (CUT&RUN) was performed using the CUTANA reagents from EpiCypher Inc., according to the manufacturer’s protocol with the following modification: Digitonin (Cell Signaling Technology, #16359L) was used at a concentration of 25 µL/mL in antibody buffer for cell permeabilization. For antibodies and dilutions used, see Supplementary Table S1. Each condition was performed on 500,000 live cells. As per the manufacturer’s protocol, 1 ng of internal *Escherichia coli* spike-in control DNA was used for all samples. DNA concentration at the conclusion of the reaction was quantified using the Quant-iT PicoGreen dsDNA assay (Thermo Fisher Scientific, #P7589). Libraries were prepared using the HyperPlus Library Preparation Kit (Roche, #07962401001) with the following modified PCR conditions: step 1: 98°C, 45 seconds; step 2: 98°C, 15 seconds; step 3: 60°C, 10 seconds; step 4: 72°C, 60 seconds; step 5: 72°C, 60 seconds; and repeat steps 2 to 4 for 12 cycles for >10 ng input DNA, 13 cycles for 5 to 10 ng input DNA, or 15 cycles for <5 ng input DNA. Libraries were analyzed for insert size distribution by the 2100 BioAnalyzer High Sensitivity kit (Agilent Technologies), 4200 TapeStation D1000 High Sensitivity ScreenTape assay (Agilent Technologies; RRID: SCR_018435), or 5300 Fragment Analyzer NGS fragment kit (Agilent Technologies; RRID: SCR_019411). Libraries were sequenced on a NextSeq (Illumina; RRID: SCR_016381) with 10 million paired-end 75–base pair reads per condition. Paired-end reads (2 × 75) were obtained from all samples. All samples were trimmed for adapters by trim_galore (version 0.4.4, “-paired -clip_R1 15 -clip_R2 15”; RRID: SCR_011847) and aligned to the human genome hg38 (Gencode GRCh38.p12) by BWA (version 0.7.12-r1039, default parameter; RRID: SCR_010910; ref. 26), and duplicated reads were then marked with biobambam2 (version 2.0.87; RRID: SCR_003308; ref. 36). BAM files were converted into bigWig coverage tracks by deepTools (version 3.5.6, default parameters; RRID: SCR_016366; ref. 37). We observed reasonable peaks on IGV (version 2.19.1; RRID: SCR_011793; ref. 38).

### High-throughput chromosome conformation capture

One million cells from D425, D458, or HDMB03 (RRID: CVCL_S506) were used for each reaction. High-throughput chromosome conformation capture (Hi-C) libraries were generated using the Arima Hi-C+ kit (Arima Genomics, #A101020) according to the manufacturer’s protocol. Chromatin cross-linking was performed using 2% formaldehyde (Sigma-Aldrich, F8775) for 10 minutes, followed by quenching with Arima Stop Solution 1 at a final concentration of 8.7%. Hi-C data were generated using the Arima-Hi-C+ kit (P/N A101020), according to the manufacturer’s protocols. Hi-C DNA was sheared using the Covaris M220 Focused-ultrasonicator (RRID: SCR_027073; peak incidence power (PIP): 50 W, duty factor (DF): 25%, cycles per burst (CPB): 200, treatment time: 65 seconds, setpoint temperature: 6°C) in 100 μL cold Elution Buffer. Fragments were size-selected to 200 bp to 600 bp prior to biotin enrichment. Before library construction, size-selected fragments were incubated at room temperature for 15 minutes with Dynabeads MyOne Streptavidin T1 beads (Invitrogen, 65,601), which were previously washed 3 times with 200 µl of Binding Buffer (10 mmol/L Tris-HCl, pH 7.5; 1 mmol/L EDTA; and 2 mol/L NaCl). Bead-bound samples were resuspended in 50 µl of Elution Buffer for library preparation using the KAPA HyperPrep Kit (Roche Sequencing Solutions, KK8505) and Integrated DNA Technologies for Illumina TruSeq DNA Indexes (Illumina, 20022370), with six amplification cycles and DNA cleanup performed using 0.85X AMPure XP Beads (Beckman Coulter, A63881). Libraries were sequenced as 151 bp paired-end reads on the Illumina NovaSeq X Plus system (RRID: SCR_024568).

### H3K27ac Hi-C followed by chromatin immunoprecipitation

Chromatin cross-linking was performed using 2% formaldehyde (Sigma-Aldrich, F8775) for 10 minutes, followed by quenching with Arima Stop Solution 1 at a final concentration of 8.7%. The assay was performed on four million HDMB03-Cas12 or D458-Cas9 cells and on three million D425-Cas9 cells. Hi-C followed by chromatin immunoprecipitation (Hi-ChIP) data were generated using the Arima-Hi-C+ kit (P/N A101020), according to the manufacturer’s protocol. Chromatin was sheared using the Covaris M220 Focused-ultrasonicator (RRID: SCR_027073; PIP: 75 W, DF: 10%, CPB: 200, treatment time: 240–260 seconds, and setpoint temperature: 7°C) in 130 μL cold CS buffer (10 mmol/L Tris-HCl, pH 8.0, 0.1% SDS). Sheared chromatin was precleared at 4°C for 1 hour on a rotator by adding 880 μL of cold MR1 Buffer [10 mmol/L Tris-HCl (pH 8.0), 159 mmol/L NaCl, 1.14 mmol/L EDTA, 1.14% Triton X-100, 0.1% SDS, 0.114% sodium deoxycholate, and 1.14% Protease Inhibitor Cocktail] and 30μL of Protein A Beads (Invitrogen, 10002D). Subsequently, each sample was incubated overnight at 4°C on a rotator with the H3K27ac antibody (Active Motif, #91193; RRID: AB_2793797) at an antibody:shearing yield ratio of 0.2. The shearing yield concentration was calculated from 10 µL of the sample taken after chromatin shearing. This aliquot was utilized as an input control in the subsequent analysis. Thereafter, each sample was incubated at 4°C for 4 hours on a rotator with Protein A Beads (Invitrogen, 10002D) previously blocked overnight with R2 Buffer [10 mmol/L Tris-HCl (pH 8.0), 140 mmol/L NaCl, 1 mmol/L EDTA, 1% Triton X-100, 0.1% SDS, 0.1% sodium deoxycholate, 1% protease inhibitor cocktail, and 5 mg/mL BSA]. Bead-bound enriched chromatin underwent several washes with different buffers to remove nonspecific fragments on a thermomixer (RRID: SCR_025397) at 4°C for 3 minutes with 1,000 rpm shaking. Respectively, three R1 buffer washes [10 mmol/L Tris-HCl (pH 8.0), 140 mmol/L NaCl, 1 mmol/L EDTA, 1% Triton X-100, 0.1% SDS, 0.1% sodium deoxycholate, and 1% protease inhibitor cocktail], two R3 buffer washes [10 mmol/L Tris-HCl (pH 8.0), 300 mmol/L NaCl, 1 mmol/L EDTA, 1% Triton X-100, 0.1% SDS, and 0.1% sodium deoxycholate), one LC Buffer wash [10 mmol/L Tris-HCl (pH 8.0), 150 mmol/L lithium chloride, 1 mmol/L EDTA, 0.5% IGEPAL CO-630, and 0.1% sodium deoxycholate], and two low EDTA TE (LTE) buffer washes [10 mmol/L Tris-HCl (pH 8.0), 0.1 mmol/L EDTA) were performed. Reverse cross-linking was conducted according to the manufacturer’s protocol. Biotin enrichment was performed using 100 μL of Arima Enrichment Beads prior to library construction. Libraries were prepared as described in the Arima-Hi-C + Hi-ChIP Library Preparation protocol, using the Swift Accel-NGS 2S Plus DNA Library Kit (Integrated DNA Technologies, 10009878), KAPA Library Amplification Kit (Roche Sequencing Solutions, 26148), and Swift Single Indexes, with 10 to 13 amplification cycles. Libraries were sequenced as 151 bp paired-end reads on the Illumina NovaSeq X Plus system (RRID: SCR_024568).

### Chromatin interaction mapping via Hi-ChIP and Hi-C

Paired-end Hi-ChIP and Hi-C sequencing data were processed using HiC-Pro (version 2.11.1; RRID: SCR_017643; ref. 39). To improve computational efficiency, FASTQ files were split into 30-million-read-pair subsets and processed in parallel. Each read end was independently aligned to the human genome (GRCh38) with Bowtie 2 (version 2.3.5.1; RRID: SCR_016368; ref. 40) using sensitive alignment parameters optimized for both global (*-very-sensitive -L 30 -score-min L,-0.6,-0.2 -end-to-end -reorder*) and local (*-very-sensitive -L 20 -score-min L,-0.6,-0.2 -end-to-end -reorder*) mapping. Reads with MAPQ <10, multimapped reads, and PCR duplicates were removed. Valid read pairs generated by HiC-Pro were subsequently converted into *.hic* format contact maps using the hicpro2juicebox.sh utility (RRID: SCR_021172).

### Coimmunoprecipitation

#### Nuclear extract (D425)

At least 20 million cells were resuspended in hypotonic Buffer A (20 mmol/L HEPES pH 7.4; 10 mmol/L KCl; and 0.2 mmol/L EDTA, with protease inhibitors) and swelled on ice for 10 minutes. Cell membranes were subsequently lysed by adding 0.6% NP-40, vortexing, and incubating on ice for 1 minute. The nuclei were collected by centrifugation for 1 minute at 16,000 × *g*, and the cytoplasmic lysate was discarded. The nuclei were then lysed in immunoprecipitation (IP) buffer supplemented with nuclease [20 mmol/L phosphobuffer pH 7.5; 150 mmol/L NaCl; 10% glycerol; 0.2% Triton X-100; protease inhibitors; 2.5 μL/mL Pierce Universal Nuclease (Thermo Fisher Scientific, #88700)] and placed on a rotary mixer for 30 minutes at 4°C. To ensure chromatin solubilization by nucleases, lysates were then incubated at room temperature for 30 minutes with frequent pipetting. Lysates were then cleared by centrifugation at 16,000 × *g* for 10 minutes.

#### Whole-cell extract (D458, SJMB016880, and HDMB03)

At least 20 million cells were directly lysed in IP buffer with nuclease [20 mmol/L phosphobuffer pH 7.5; 150 mmol/L NaCl; 10% glycerol; 0.2% Triton X-100; protease inhibitors; and 2.5 μL/mL Pierce Universal Nuclease (Thermo Fisher Scientific, #88700)]. Lysates were incubated on a rotary mixer for 20 minutes at 4°C, then incubated at room temperature for 30 minutes with frequent pipetting, and cleared by centrifugation at 16,000 × *g* for 10 minutes.

#### Immunoprecipitation of nuclear proteins

Lysates (nuclear or total) were quantified using a BCA kit (Thermo Fisher Scientific) and diluted to a final concentration of 1 mg/mL using IP buffer. Lysate (500–1,000 µg) was precleared with Protein G Dynabeads (Thermo Fisher Scientific, #10003D) and resuspended in IP buffer. Lysates were precleared on a rotary mixer for 1.5 hours at 4°C. Inputs (5% of total volume) were saved and boiled with lithium dodecyl sulfate (LDS) for subsequent immunoblotting analyses. Precleared lysate was rotated overnight at 4°C with the indicated antibodies (Supplementary Table S1). Antibody–protein conjugates were purified from lysates by incubation with Protein G Dynabeads (Thermo Fisher Scientific, #10003D) for 15 minutes at 20°C. Bead–antibody–protein conjugates were washed 3 times with cold IP buffer and rotated for 3 minutes at 4°C each time. Bead–antibody–protein conjugates were eluted from beads with 1× LDS at 95°C for 10 minutes.

#### Immunoblotting analysis

Inputs and IP eluates were run on 3% to 8% tris-acetate gels and transferred onto 0.45-µm nitrocellulose membranes. Membranes were blocked with 5% milk in TBS-T for one hour and then incubated with primary antibodies (Supplementary Table S1) overnight at 4°C. Membranes were washed with TBS-T 3 times for 5 minutes and then incubated with peroxidase-conjugated light-chain-specific secondary antibodies for 1 hour at room temperature. Membranes were then washed with TBS-T 3 times and imaged with the LICOR Odyssey XF Imaging System (RRID: SCR_023765) and analyzed with ImageStudio software (RRID: SCR_015795).

## Results

### Coamplification of *MYC* and *OTX2* on ecDNA in G3-MB

**Figure 1.**
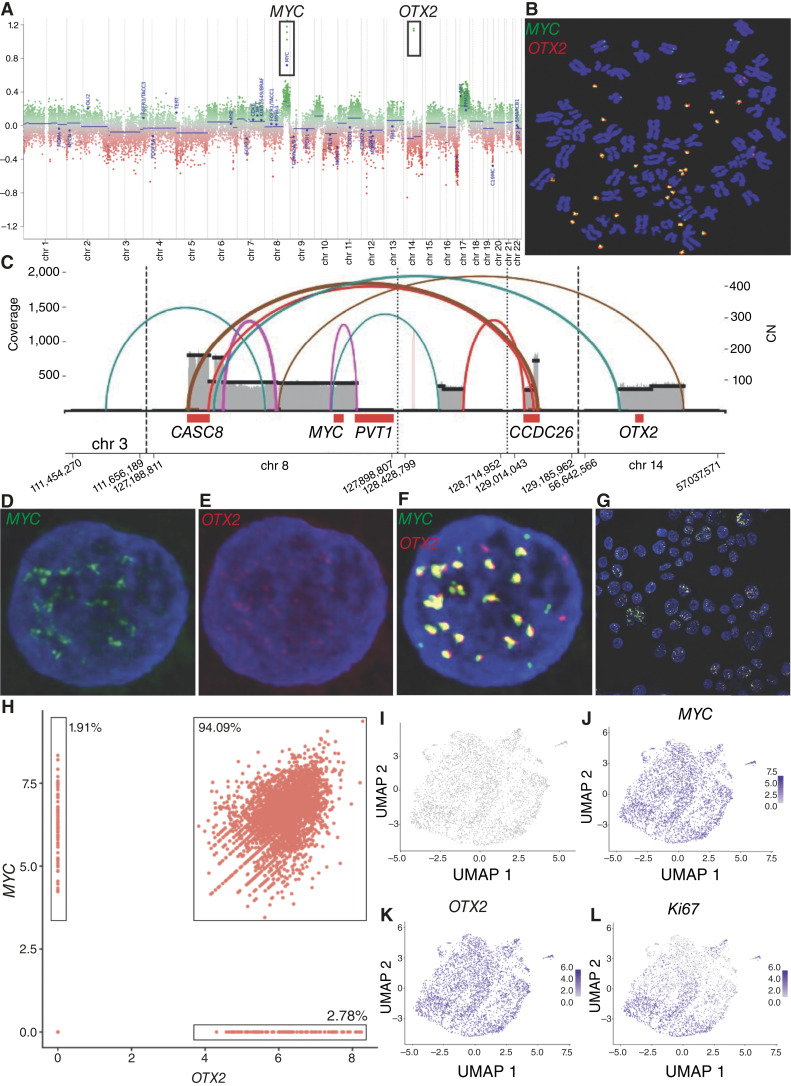
ecDNAs in D425 cells exhibit coamplification of *MYC* and *OTX2*. **A,** Copy-number variation plot derived from EPIC 850k methylation sequencing of the D425 cell line (41). Green represents amplification events, whereas red represents deletion events. **B,** Representative DNA metaphase FISH imaging of the D425 cell line *MYC* (green) and *OTX2* (red). Yellow signals indicate colocalization of probes. **C,** AmpliconArchitect reconstruction of the D425 DM (gray coverage track). Arcs represent structural variant breakpoints and orientation (red, expected orientation; brown, everted pair; cyan, both reads map to forward strand [tip to tip]; purple, both reads map to reverse strand [tail to tail]). Red bars identify selected regulatory and protein-coding gene regions amplified within the DM. CN, inferred copy number; coverage, depth of sequencing. **D** and **E,** Representative RNA interphase FISH imaging for the D425 cell line targeting (**D**) *MYC* (green) and (**E**) *OTX2* (red). **F,** Representative DNA interphase FISH imaging of the D425 cell line targeting *MYC* (green) and *OTX2* (red). **G,** Wide-field FISH imaging targeting *MYC* (green) and *OTX2* (red). **H,** Dot plot showing gene expression levels of *MYC* (*y*-axis) and *OTX2* (*x*-axis) in each single cell from the D425 cell line. The percentage for each cell population is annotated. **I,** Uniform Manifold Approximation and Projection for Dimension Reduction (UMAP) plot for the D425 cell line. **J**-**L,** Gene expression levels of (**J**) *MYC*, (**K**) *OTX2*, or (**L**) *Ki-67* transcription at the single-cell level on UMAP plots in the D425 cell line.

Genome-wide measurements of DNA methylation using the EPIC 850k array (41) identified focal coamplification of the *MYC* and *OTX2* genes on chromosomes 8 and 14, respectively, in the G3-MB cell line D425 (Fig. 1A; ref. 18). This cell line also harbors a terminal deletion of the long arm of chromosome 10 (del10q), a single-copy loss of the short arm of chromosome 17 (i17q, which contains *TP53*), and a duplication of the long arm. The intact chromosome 17p contains a missense mutation in *TP53*, rendering D425 *TP53*-null. DNA FISH imaging revealed that *MYC* and *OTX2* amplification occurred within ecDNA (Fig. 1B). Reconstruction of the D425 ecDNA using AmpliconArchitect (42) confirmed the coamplification of *MYC* and *OTX2* and resolved the complex rearrangement of the 8q24 regulatory DNA region surrounding *MYC* (Fig. 1C). This complexity could not be inferred by the EPIC 850k methylation array alone. The D425 ecDNA harbors amplifications of the *PVT1* promoter but not the gene body, the established *MYC* enhancer, and lncRNA *CCDC26* (43), as well as many cell context-specific *MYC* enhancers 5’ to the *MYC* promoter (43), including cancer susceptibility candidate 8 [*CASC8* (Fig. 1C)].

Sequential RNA and DNA FISH imaging of interphase D425 cells revealed that both *MYC* (Fig. 1D) and *OTX2* (Fig. 1E) RNA expression arose mainly from ecDNA-specific rather than endogenous chromosomal loci (Fig. 1F), suggesting that ecDNAs are the main drivers of *MYC* and *OTX2* transcription. Wide-field FISH microscopy demonstrated that D425 cells harbor different copy numbers of ecDNAs per cell (Fig. 1G), as expected from the random segregation of DMs into daughter cells, resulting in intratumor copy-number heterogeneity (44). To determine the role of copy-number diversity in *MYC* and *OTX2* transcription in individual cells, we performed single-cell RNA sequencing of D425 cells. Most cells displayed uniformly high *MYC* and *OTX2* expression despite ecDNA copy-number heterogeneity (Fig. 1H–L). Dimensional reduction showed no clustering of high/low *MYC-* or *OTX2-*expressing cells (Fig. 1J and K), but there was clustering by Ki-67, indicating that the results were not due to cell-cycle effects (Fig. 1L). These results point to the role of active regulatory mechanisms that titrate *MYC* expression within the cell population, agnostic of ecDNA copy number.

### D425 cells are dependent on MYC but not OTX2

**Figure 2.**
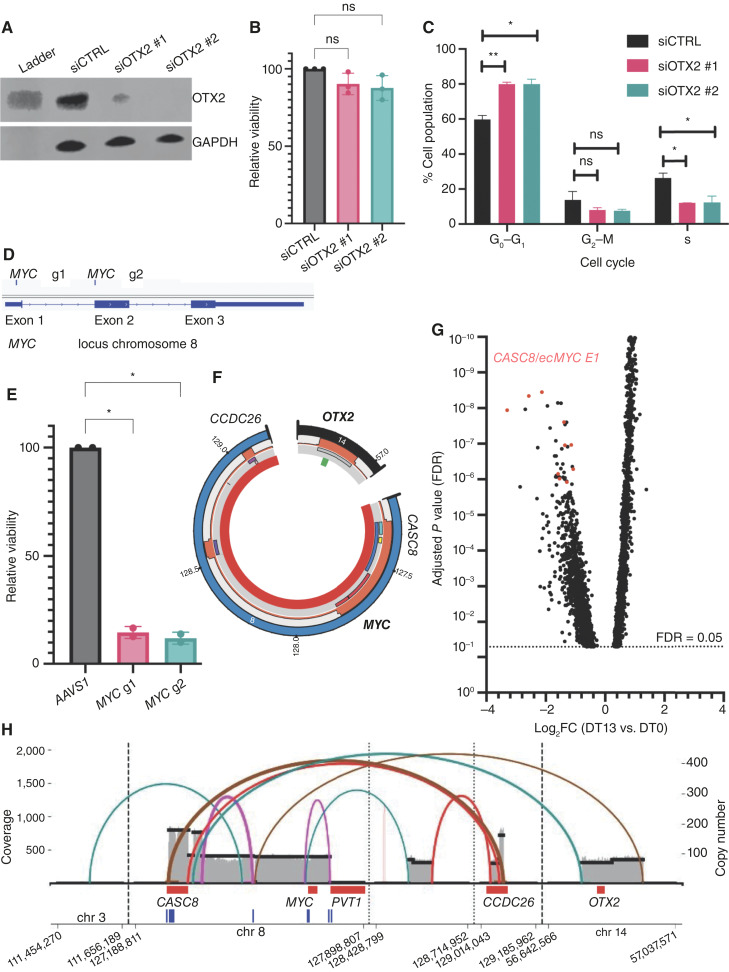
D425 relies on *MYC* but not *OTX2* for proliferation and survival. **A,** Immunoblotting for OTX2 in D425 at 96 hours after siRNA transfection against OTX2. **B,** D425 viability at 96 hours after OTX2 knockdown by siRNA. **C,** Flow cytometry–based cell-cycle analysis of the D425 cell line at 96 hours after OTX2 siRNA knockdown. *, *P* <0.05; **, *P* <0.005. **D,** Schematic showing the locations of the two *MYC* promoter sgRNAs for the D425 dCas9-KRAB system validation in relation to the *MYC* locus on chromosome 8. **E,** D425 viability 72 hours after infection with sgRNAs targeting the *MYC* promoter, compared with *AAVS1* safe harbor targeting chromosome 19 as a negative control. *, *P* <0.05. **F,** CIRCOS plot of the Episomizer reconstruction of the D425 ecDNA DM overlaid with the CRISPRi screening library directed to chromosome 8, depicted by the red semicircle, and the *OTX2* promoter, depicted by the green semicircle. The outermost circle represents each chromosome amplified on the D425 ecDNA: chromosome 8 (blue) and chromosome 14 (black). The orange track represents the WGS coverage track for each chromosome, indicating the regions amplified on the ecDNA. The individual amplified fragments are represented by colored bars within the gray circle. **G,** Volcano plot highlighting the enrichment of significantly depleted sgRNAs targeting the *ecMYC E1* region at the conclusion of the *MYC* regulatory region sgRNA screen of the D425 ecDNA. **H,** Locations of the most depleted sgRNAs (blue ticks) mapped to the D425 AmpliconArchitect reconstruction. FC, fold change.

We consulted the Cancer Dependency Map Project (DepMap) portal (45) to investigate the underlying dependency for D425 cell proliferation on *MYC* and *OTX2*. The DepMap provides Chronos scores from genome-wide CRISPR–Cas9 knockout screens for each cell line, indicating the necessity of each gene for viability and proliferation (46). A negative Chronos score indicates a dependency on a specific protein, whereas a positive score indicates a survival advantage when that protein is lost (46). The DepMap CRISPR knockout screen for D425 showed a weak positive Chronos score of +0.17 for *OTX2*, implying that OTX2 is not essential for D425 tumor cell survival or proliferation (Supplementary Fig. S1A). A similar lack of dependency on OTX2 was observed in an additional *MYC*-amplified G3-MB cell line, D458, in a whole-genome CRISPR knockout screen generated by the DepMap, with a Chronos score of −0.33 (Supplementary Fig. S1B; ref. 45). Silencing of OTX2 protein expression in D425 cells (Fig. 2A) revealed no significant decrease in cell viability after 96 hours of knockdown (Fig. 2B), supporting the findings of the DepMap CRISPR screen (45). OTX2 knockdown increased the proportion of cells in the G_0_–G_1_ stage of the cell cycle, with a concomitant decrease in the percentage of the cell population in S or G_2_–M (Fig. 2C), suggesting that OTX2 may drive G_1_ progression and promote proliferation.

In contrast, *MYC* loss was associated with a strong negative Chronos score of −1.47, suggesting a strong dependency on MYC for cell proliferation and survival in D425 (Supplementary Fig. S1A). We leveraged dCas9-KRAB epigenetic silencing to reduce *MYC* transcription. After generating a D425 cell line stably expressing dCas9-KRAB, we targeted the dCas9-KRAB machinery to the *MYC* promoter using two independent sgRNAs (Fig. 2D; Supplementary Table S2). As control, we targeted the *AAVS1* within intron 1 of the gene encoding the protein phosphatase-1 12C regulatory subunit on chromosome 19, a “safe-harbor” locus in which disruption does not cause discernible adverse effects on cell viability or proliferation (47). Seventy-two hours after infection, there was a significant decrease in viability upon targeting the *MYC* promoter compared with the *AAVS1* safe harbor control (Fig. 2E), supporting the strong negative Chronos score observed in the DepMap portal (45). This suggests that *MYC*, but not *OTX2*, is a dependency in D425.

### *ecMYC E1* enhancer is a putative activator of *MYC* transcription in D425 cells

The dCas9-KRAB sequence-specific epigenetic silencing machinery was utilized to perform a CRISPR inhibition (CRISPRi) screen that systematically targeted the chromosome 8q24 *MYC* regulatory locus amplified within the D425 ecDNA (Fig. 2F; Supplementary Table S3). The CRISPRi library also included control sgRNAs targeting the *OTX2* and *MYC* promoters—as negative and positive controls, respectively—as well as nontargeting sgRNAs (Fig. 2F; Supplementary Table S3). Of the top 15 most depleted sgRNAs following MAGeCK analysis (32), six of them targeted a 34-kb region (Hg38: chr8:127,294,177–127,328,982) about 500 kb 5′ to the *MYC* promoter (Table 1; Fig. 2G and H). This segment is localized within a larger regulatory DNA region, specifying the long noncoding RNA *CASC8* that is only partially amplified within the ecDNA (Fig. 2H). The *CASC8* locus was recently reported to include a *MYC* enhancer in the D458 cell line (5), derived from the relapse tumor from the same patient whose primary tumor was the source of the D425 cell line (18). Reconstruction of the D458 ecDNA (Supplementary Fig. S2A) documents a larger amplification event of the 8q24 locus, mostly comprising the region 3′ to *MYC,* including the *PVT1* coding region and regulatory sequences that are not contained within the D425 ecDNA (Fig. 1C; refs. 5, 43). AmpliconArchitect reconstructions of two additional *MYC*-amplified G3-MB models, HDMB03 (Supplementary Fig. S2B) and SJMB016880 (Supplementary Fig. S2C), also showed coamplification of the *MYC* enhancer, which we termed *ecMYC E1*.

**Table 1.** Top 15 most depleted sgRNAs from D425 CRISPRi screen.

Guide RNA	Targeting coordinates (Hg38)	Target locus
4,580	chr8:127,251,768–127,251,787	*CASC8*
1,004	chr8:127,303,566–127,303,585	*CASC8*
153	chr8:129,984,302–129,984,321	*FAM49B*
8,788	chr8:127,303,500–127,303,519	*CASC8*
6,392	chr8:128,176,317–128,176,336	*PVT1* promoter
1,251	chr8:129,858,488–129,858,507	*FAM49B*
4,039	chr8:127,737,006–127,737,025	*MYC* promoter
367	chr8:127,566,416–127,566,435	N/A
1,674	chr8:126,897,326–126,897,345	*CASC8*
4,156	chr8:127,736,097–127,736,116	*MYC* promoter
591	chr8:126,525,790–126,525,809	N/A
4,770	chr8:127,029,277–127,029,296	*CASC8*
7,009	chr8:128,199,910–128,199,929	N/A
1,772	chr8:127,919,655–127,919,674	*PVT1* promoter
9,731	chr8:127,303,507–127,303,526	*CASC8*

NOTE: List of sgRNAs and locus annotation for the 15 most depleted sgRNAs at the conclusion of the CRISPRi screen.

Abbreviation: N/A, not annotated.

### *ecMYC E1* displays enhancer hallmarks in ecDNA- and HSR-positive G3-MB

**Figure 3.**
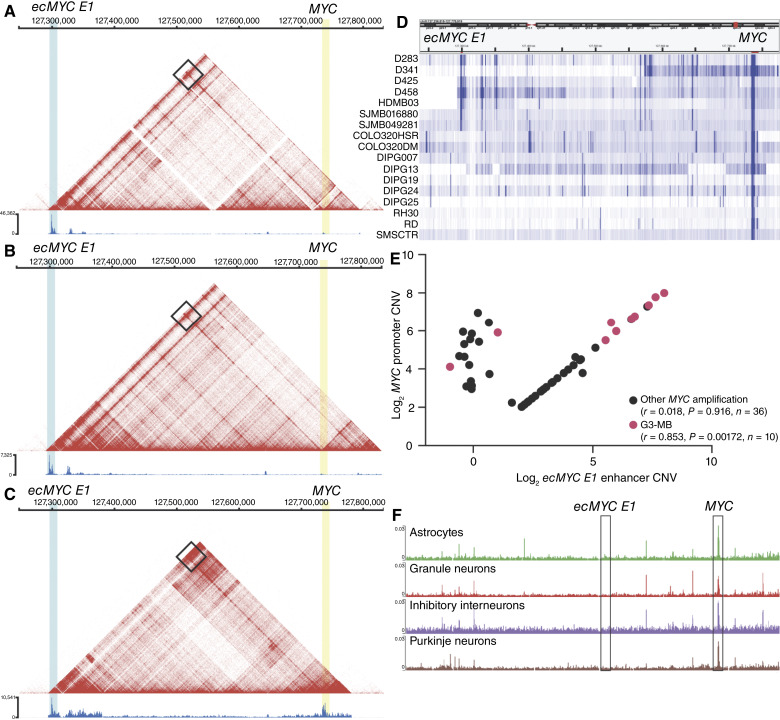
*ecMYC E1* displays canonical enhancer hallmarks in *MYC*-amplified G3-MB. **A–C,** H3K27ac-anchored Hi-ChIP contact map shows H3K27ac-mediated looping interactions between the *MYC* promoter (yellow marker) and the *ecMYC E1* locus (blue marker) (identified by the black box) along with H3K27ac occupancy by ChIP-seq (represented by the blue sequencing track) at the *ecMYC E1* locus in (**A**) D425, (**B**) D458, and (**C**) HDMB03. **D,** Aligned ATAC-seq track heatmap of *MYC*-amplified G3-MB: D283, D341, D425, D458, HDMB03, SJMB016880, and SJMB049281; non-MB *MYC*-amplified: COLO320HSR and COLO320DM; *MYC*-overexpressing DIPG: DIPG007, DIPG13, DIPG19, DIPG24, and DIPG25; and *MYC*-overexpressing solid tumors: RH30, RD, and SMSCTR. **E,** Copy-number variation for the *MYC* promoter (*y*-axis) versus copy number of the *ecMYC E1* enhancer (*x*-axis) across *MYC*-amplified cancers: G3-MB (pink dots), all other tumor types (black dots). **F,** ATAC-seq tracks from cells of the developing human cerebellum at the 8q24 locus (48).

We used data generated by the PedDep to investigate the potential enhancer signature of the *ecMYC E1* locus in G3-MB more broadly and compared it with other *MYC*-amplified and *MYC*-dependent cancer cell lines (28). Using H3K27ac-anchored Hi-ChIP sequencing, we first looked at chromatin looping interactions between the *ecMYC E1* locus and the *MYC* promoter in three G3-MB cell lines with *MYC* amplification: two ecDNA-containing cell lines, D425 (Fig. 3A) and D458 (Fig. 3B), and HDMB03 (Fig. 3C), in which *MYC* is amplified on HSRs. The H3K27ac Hi-ChIP contact maps for all three models, D425 (Fig. 3A), D458 (Fig. 3B), and HDMB03 (Fig. 3C), showed an increase in the number of reads connecting the *MYC* locus (yellow highlight) and the *ecMYC E1* locus (blue highlight), indicating recurrent looping interactions between these two regions. The *ecMYC E1* region also displayed strong H3K27ac occupancy by ChIP sequencing (ChIP-seq; Fig. 3A–C), further reinforcing the idea that this region is an active enhancer in *MYC*-amplified G3-MB. This looping interaction was confirmed by unbiased genome-wide Hi-C sequencing, which showed increased DNA–DNA contacts between the *ecMYC E1* enhancer (blue highlight) and the *MYC* promoter (yellow highlight) in two ecDNA-containing cell lines, D425 (Supplementary Fig. S3A) and D458 (Supplementary Fig. S3B), and the HSR-containing line HDMB03 (Supplementary Fig. S3C).

*ecMYC E1* displayed open chromatin identified by ATAC-seq only in *MYC*-amplified G3-MB, suggesting it is a specific enhancer for this subgroup (Fig. 3D). This included seven established G3-MB models: three with *MYC* amplified on HSRs, HDMB03 (Supplementary Fig. S4A), D283 (Supplementary Fig. S4B), and D341 (Supplementary Fig. S4C), and four with *MYC* amplified on ecDNA, D425, D458, and the tumor organoid SJMB016880 (Supplementary Fig. S4D) and SJMB049291 (Supplementary Fig. S4E). We did not detect enhancer hallmarks of open chromatin at the *ecMYC E1* locus in the two *MYC*-amplified human colorectal carcinoma cell lines: COLO320HSR and COLO320DM; five MYC-overexpressing DIPG lines: DIPG007, DIPG13, DIPG19, DIPG24, and DIPG25; or three MYC-dependent human solid tumor cell lines: RH30, RD, and SMSCTR (Fig. 3D; ref. 49). Although *ecMYC E1* was coamplified with *MYC* in four of the models (COLO320HSR, COLO320DM, DIPG007, and DIPG24), the locus did not display enhancer hallmarks of open chromatin in these lines, indicating that *MYC* transcription is driven by other mechanisms in these tumors (43).

We further queried the relationship between the *ecMYC E1* enhancer and *MYC* gene coamplification in *MYC*-amplified cancers within the DepMap and St. Jude cohorts, including both adult and pediatric samples. We defined *MYC* amplification by any tumor sample that contained six or more copies of the *MYC* gene, regardless of its ecDNA or HSR status. This analysis revealed a strong Pearson’s correlation for the coamplification of *ecMYC E1* and the *MYC* promoter in G3-MB (*r* = 0.853, *P* = 0.00172, *n* = 10) compared with all other pediatric and adult *MYC*-amplified tumors (*r* = 0.018, *P* = 0.916, *n* = 36; Fig. 3E), supporting the conclusion that *ecMYC E1* is a hallmark enhancer in *MYC*-amplified G3-MB. To determine if this enhancer is engaged during normal embryogenesis, we analyzed ATAC-seq data of cells from the developing human fetal cerebellum, which failed to reveal open chromatin for *ecMYC E1*(Fig. 3F; ref. 48), including the proposed cell of origin for G3-MB, cerebellar granule neuronal progenitors (Fig. 3F; ref. 2). We found no evidence of H3K4me1 enrichment (a marker for poised or active enhancers; ref. 50) at the *ecMYC E1* locus in mouse epithelial stem cells or in mouse neural progenitor cells (Supplementary Fig. S5A and S5B; bioRxiv 2021.03.17.435905; GSE160890). This further supports that this enhancer functions preferentially in *MYC*-amplified G3-MB.

### *ecMYC E1* activates *MYC* transcription in ecDNA-positive G3-MB

**Figure 4.**
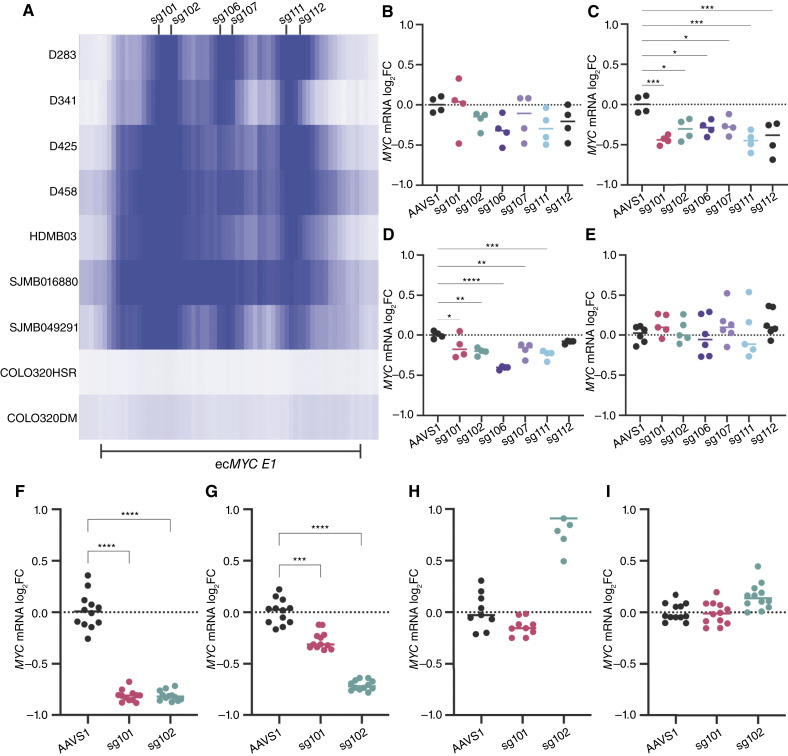
*ecMYC E1* activates *MYC* transcription in ecDNA-positive G3-MB. **A,** ATAC-seq open chromatin heatmap at the *ecMYC E1* enhancer locus (Hg38 chr8: 127,298,500–127,300,500) in *MYC*-amplified models. The top four tracks represent *MYC*-amplified G3-MB cell lines developed at Duke University. The fifth track represents the HDMB03 HSR-driven G3-MB cell line. The sixth and seventh tracks represent ecDNA+ patient-derived tumor organoid G3-MB models. The bottom two tracks represent the *MYC*-amplified cell line COLO320 in the HSR and DM forms, respectively. Markings above the first track represent the region targeted by each respective annotated sgRNA. **B–E,** RT-qPCR for *MYC* mRNA transcription after targeted silencing of the *ecMYC E1* enhancer in the D425 cell line at (**B**) 0, (**C**) 3, (**D**) 7, and (**E**) 13 doublings. *, *P* <0.05; **, *P* <0.005; ***, *P* <0.0005; ****, *P* <0.00005. **F** and **G,** RT-qPCR for *MYC* mRNA transcription after targeted silencing of the *ecMYC E1* enhancer in the (**F**) D458 cell line and (**G**) SJMB016880 tumor organoid at 3 doublings after infection. *** *P* <0.0005; **** *P* <0.00005. **H** and **I,** RT-qPCR for *MYC* mRNA transcription after targeted silencing of the *ecMYC E1* enhancer in the (**H**) D458 cell line and (**I**) SJMB016880 tumor organoid at 13 doublings after infection. FC, fold change.

Chromatin accessibility analyses to validate *ecMYC E1* as an activator of *MYC* transcription identified three ATAC-seq peaks in a 1,500–base pair window that were present in all *MYC*-amplified G3-MB but not in the isogenic *MYC*-amplified colorectal carcinoma cell lines COLO320HSR and COLO320DM at the hg38 coordinates chr8: 127,299,000–127,300,500 (Fig. 4A). Using the CRISPick sgRNA design tool (30, 51), we designed individual sgRNAs targeting opposite DNA strands of the *ecMYC E1* to position the dCas9-KRAB silencing module at the apex of each peak (Supplementary Table S2). The expression of sgRNAs cloned into an ametrine-containing vector was monitored by flow cytometry for marker expression. Infected D425 cells were sorted for the top 10% of ametrine-expressing cells 48 hours after infection (designated doubling time 0, DT0). RT-qPCR at DT0 showed a slight decrease in *MYC* mRNA expression, which became more pronounced at DT3 and DT7 (3 and 7 doubling times after infection, respectively; Fig. 4B–D). However, we observed a return to baseline *MYC* mRNA expression at DT13 (Fig. 4E), suggesting that D425 cells are compensating for the loss of *MYC* transcription.

To validate the data from the D425 cell line, we used two additional *MYC* ecDNA-amplified G3-MB models: one DM-positive cell line, D458 (5), and one DM-positive tumor organoid, SJMB016880 (Supplementary Fig. S2C and S2D). Both models contained *ecMYC E1* within the complex amplicon (Supplementary Fig. S2A and S2C; ref. 5). Targeted silencing of the *ecMYC E1* locus using sg101 or sg102 led to a significant decrease in *MYC* mRNA expression in the D458 and SJMB016880 G3-MB models at DT3 (Fig. 4F and G). As seen in the D425 cell line, following silencing, we observed a return to baseline or even greater *MYC* mRNA expression in the D458 and SJMB016880 G3-MB models at DT13 (Fig. 4H and I).

Previous investigations have implicated a relationship between the *PVT1* promoter and *MYC* transcription (52, 53). All *MYC*-amplified G3-MB models tested in this article demonstrated open chromatin at the *PVT1* promoter locus (Supplementary Fig. S6A), but none of the models displayed the *PVT1*-*MYC* fusion that stabilizes oncogenic mRNA (Supplementary Fig. S6B; ref. 53). We targeted the dCas9-KRAB machinery to the *PVT1* promoter with four different sgRNAs (Supplementary Fig. S6C). This resulted in no significant decrease in *MYC* mRNA in the D458 cell line (Supplementary Fig. S6D), consistent with the data from Chapman and colleagues (5), and a minimal decrease in the SJMB016880 tumor organoid model (Supplementary Fig. S6E). Both models displayed a significant loss in *MYC* transcription following *ecMYC E1* silencing at the same time point (Supplementary Fig. S6D and S6E).

A recent report identified a transposable element on chromosome 3 (Hg38: chr3:111,555,200–111,556,000), termed ecDNA interacting element 14 (EIE14), as an activator of *MYC* transcription in the COLO320DM cell line (54). This ∼1 kb region is inserted into the chromosome 8 locus in 46% of nondisease individuals and is amplified on the ecDNA or HSR of all *MYC*-amplified G3-MB lines used in this study (Fig. 1C; Supplementary Fig. S2A–S2C). There was significantly less occupancy of H3K27ac at the EIE14 locus compared with the *ecMYC E1* enhancer or *MYC* promoter in ecDNA or HSR-containing G3-MB models, indicating that this region is not an active enhancer in G3-MB (Supplementary Fig. S7A and S7B).

### ecDNA-positive G3-MBs compensate for *MYC* silencing by increasing ecDNA copy number

**Figure 5.**
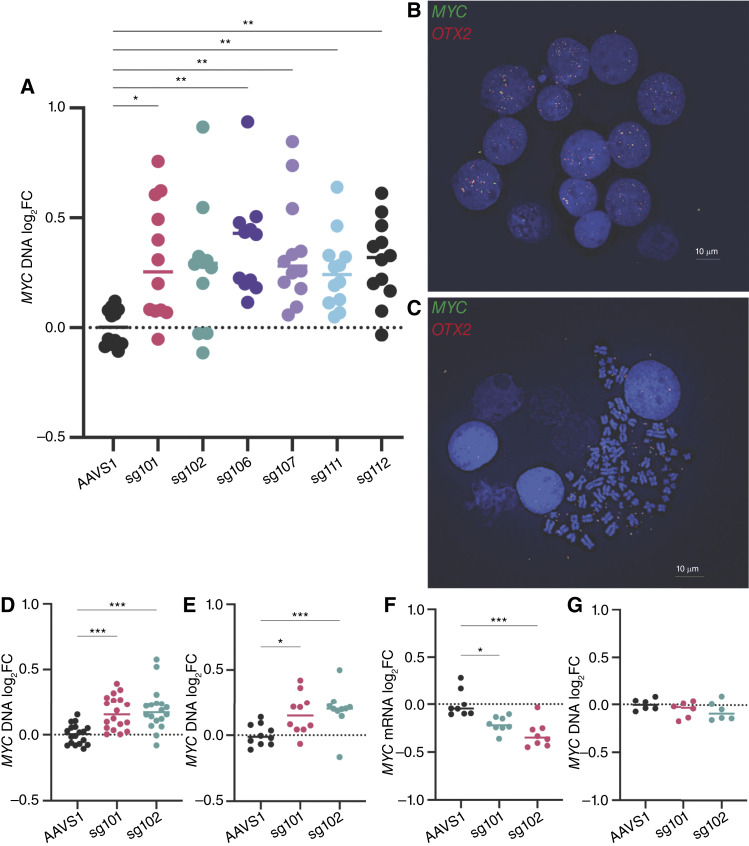
ecDNA-positive G3-MB compensates for *MYC* silencing by increasing ecDNA copy number. **A,** DNA qPCR for *MYC* copy number in the D425 cell line at DT13 following *ecMYC E1* silencing. *, *P* <0.05; **, *P* <0.005. **B,** Representative DNA FISH image for *MYC* (green) and *OTX2* (red) of the D425 cell line with dCas9-KRAB targeted to *ecMYC E1* using sg102 after 13 doubling times (DT13). The scale bar in the lower right-hand corner indicates a length of 10 µm. **C,** Representative DNA FISH image for *MYC* (green) and *OTX2* (red) of the D425 cell line with dCas9-KRAB targeted to the *AAVS1* safe-harbor locus at DT13. The scale bar in the lower right-hand corner indicates a length of 10 µm. **D** and **E,** DNA qPCR for *MYC* copy number in the (**D**) D458 cell line and (**E**) SJMB016880 umor organoid at DT13 following *ecMYC E1* silencing. *, *P* <0.05; ***, *P* <0.0005. **F,** RT-qPCR for *MYC* mRNA transcription after targeted silencing of the *ecMYC E1* enhancer in the HSR-containing HDMB03 cell line at DT13.  *, *P* <0.05; ***, *P* <0.0005. **G,** DNA qPCR for *MYC* copy number in the HSR-containing HDMB03 cell line at DT13 following *ecMYC E1* silencing. FC, fold change.

It has long been known that DMs can undergo episomal amplification to bypass conditions that would otherwise lead to their negative selection. In classic experiments, Alt, Schimke, and their collaborators demonstrated that tumor cells containing DMs that amplified the *DHFR* gene responded to the chemotherapeutic drug methotrexate by selecting for tumor cells with increased numbers of *DHFR*-containing ecDNAs (55). Similarly, a recent study showed that cells with *FGFR2* amplification exhibit increased ecDNA copy number following targeted FGFR2 inhibition, underscoring the role of oncogenes in providing a proliferative advantage and the ability of randomly segregated episomal elements to rapidly confer a survival benefit (10). Reasoning that the ecDNA-positive G3-MB cells might respond through a similar mechanism, we performed DNA copy-number qPCR in the D425 cell line at DT13 following silencing, which documented a significant increase in ecDNA copy number for each *ecMYC E1*-targeted condition compared with the *AAVS1* control (Fig. 5A). DNA FISH imaging confirmed a significant increase in the number of ecDNAs on a cell-by-cell basis after *ecMYC E1* silencing by sg102 compared with an sgRNA directed to the *AAVS1* safe harbor control locus (Fig. 5B and C). AmpliconArchitect reconstruction of the D425 ecDNA after *ecMYC E1*- or *AAVS1*-targeting showed that the ecDNAs were identical in both conditions at DT13, suggesting that compensation was not due to the generation or selection of new ecDNA species (Supplementary Fig. S8A and S8B). We performed the same DNA copy-number qPCR at DT13 in the D458 cell line and in the SJMB016880 tumor organoid G3-MB models and observed a significant increase in ecDNA copy number in silenced conditions compared with the *AAVS1* safe-harbor targeting condition (Fig. 5D and E). However, when the same experiment was performed in the HDMB03 G3-MB cell line in which *MYC* is amplified on an HSR, *MYC* mRNA transcription remained significantly reduced at DT13 (Fig. 5F), and *MYC* copy number was unchanged (Fig. 5G). Therefore, in ecDNA- but not HSR-containing G3-MB models, *MYC* mRNA expression decreased after *ecMYC E1* silencing, but this loss was compensated for over time by increases in ecDNA copy number.

### BRD4andNeuroD1bind* ecMYC E1* to activate *MYC* transcription

**Figure 6.**
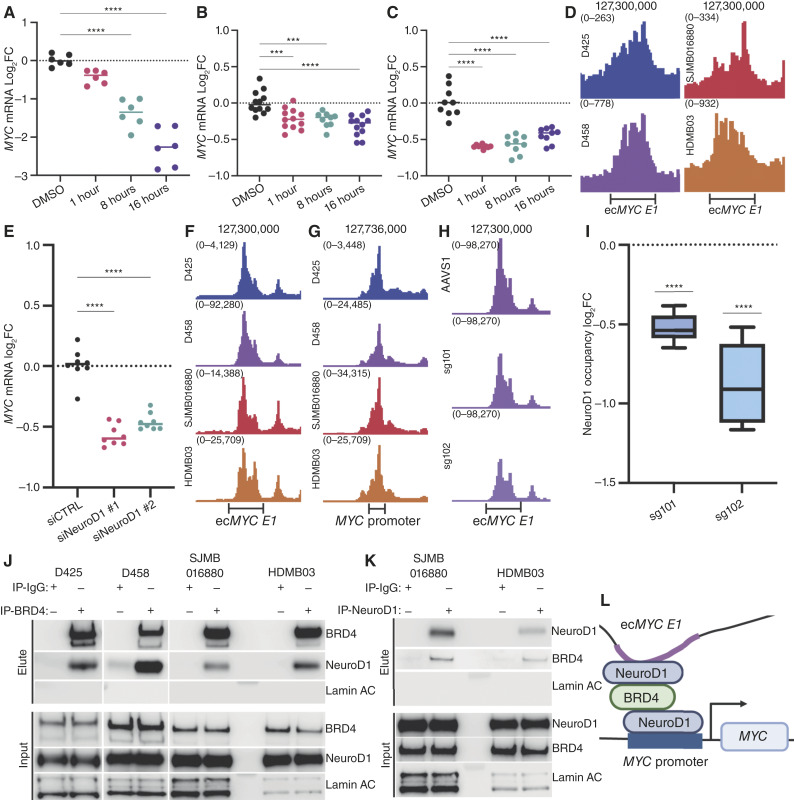
BRD4 and NeuroD1 bind *ecMYC E1* to activate *MYC* transcription. **A–C,** RT-qPCR for *MYC* mRNA transcription following 100 nmol/L SJ432 treatment in the (**A**) D425 and (**B**) D458 cell lines and the (**C**) SJMB016880 tumor organoid. **D,** CUT&RUN targeting BRD4 at the *ecMYC E1* enhancer locus in the D425 (blue), D458 (purple), SJMB016880 (red), and HDMB03 (orange) G3-MB models. **E,** RT-qPCR for *MYC* mRNA transcription following NeuroD1 silencing in the D458 cell line. **F** and **G,** ChIP-seq targeting NeuroD1 at the (**F**) *ecMYC E1* enhancer locus or (**G**) *MYC* promoter in the D425 (top row), D458 (second row), SJMB016880 (third row), and HDMB03 (bottom row) G3-MB cell models. **H,** ChIP-seq targeting NeuroD1 at the *ecMYC E1* enhancer locus in the D458 cell line at DT3 after silencing (sg101 or sg102) or by nontargeting control (AAVS1). **I,** Log_2_ fold change (FC) in NeuroD1 occupancy at the *ecMYC E1* enhancer at DT3 following silencing compared with the AAVS1 nontargeting control. **J** and **K,** Co-IP pulldown for (**J**) BRD4 or (**K**) NeuroD1. Lamin A/C: noninteracting control. **L,** Proposed mechanism for *ecMYC E1* activation of *MYC* transcription through the binding of BRD4 and NeuroD1 at the enhancer and looping to the *MYC* promoter in *MYC*-amplified G3-MB. *, *P* <0.05; **, *P* <0.005; ***, *P* <0.0005; ****, *P* <0.00005. Created in BioRender. Friske, J. (2026) https://BioRender.com/9hm4nmn.

Previous reports highlighted the role of BRD4 as a regulator of *MYC* transcription in leukemia and G3-MB (56, 57). Treating the D425, D458, and SJMB016880 G3-MB models with the BD2-specific BRD4 inhibitor, SJ432, led to a significant reduction in *MYC* mRNA transcription in as little as one hour after treatment compared with the DMSO-treated control (Fig. 6A–C; ref. 29). This reduction continued through the 8- and 16-hour time points (Fig. 6A–C). CUT&RUN sequencing displayed enrichment of BRD4 at the *ecMYC E1* enhancer and *MYC* promoter loci in all four tested *MYC*-amplified G3-MB models (3 ecDNA+ and 1 HSR+; Fig. 6D; Supplementary Fig. S9), supporting the role of BRD4 as a cofactor for *MYC* transcription in G3-MB, amplifying *MYC* in ecDNA and HSR.

The transcription factor NeuroD1 was identified as a selective dependency in *MYC*-amplified G3-MB, in which dependency on NeuroD1 expression correlated with *MYC* copy number (Pearson *r* = −0.472; *P* = 0.0423) (Supplementary Fig. S10A and S10B; ref. 28). NeuroD1 plays a central role in the development of the central nervous system and promotes neuronal survival in various brain regions, including the cerebellum (58). Publicly available NeuroD1 ChIP-seq data in the *MYC*-amplified G3-MB cell lines D283 and D341 revealed its binding to the *ecMYC E1* enhancer (Supplementary Fig. S10C). siRNA-mediated knockdown of NeuroD1 in D458 cells documented a significant reduction in *MYC* mRNA transcription after 48 hours of treatment (Fig. 6E; Supplementary Fig. S11). Subsequent ChIP-seq experiments indicated that NeuroD1 binds both to the *ecMYC E1* enhancer (Fig. 6F) and the *MYC* promoter (Fig. 6G) in *MYC*-amplified G3-MB: D425, D458, SJMB016880, and HDMB03. A significant reduction in NeuroD1 occupancy was observed at the *ecMYC E1* enhancer locus in the D458 G3-MB cell line three doublings after *ecMYC E1* silencing (Fig. 6H and I), supporting its role in activating *MYC* transcription. Coimmunoprecipitation (Co-IP) assays revealed a direct, reciprocal interaction between NeuroD1 and BRD4 in all tested *MYC*-amplified G3-MB models (Fig. 6J and K). In conclusion, we propose that a transcriptional regulatory complex containing NeuroD1 and BRD4 binds to the *ecMYC E1* enhancer specifically in *MYC*-amplified G3-MB to activate *MYC* transcription and drive proliferation (Fig. 6L).

## Discussion

Gene copy-number variation identified from methylation array analysis revealed *MYC* amplification in the G3-MB cell line D425 (18), a phenomenon we subsequently documented in additional G3-MB cell lines and organoids. Reconstruction using two methods, including AmpliconArchitect (42) and Episomizer (22), indicated that *MYC* and *OTX2* were coamplified on the same amplicon and coordinately expressed from ecDNA loci. Aberrant OTX2 activity promotes cell-cycle progression and inhibits neuronal differentiation—hallmarks of pediatric malignant brain tumors (59). As such, *OTX2* has been implicated as an oncogene in medulloblastoma (59). Although its loss did not affect cell viability, its absence increased the proportion of tumor cells in the G_0_–G_1_-phase, supporting a role for OTX2 in promoting G_1_ progression and increased proliferation (59). In contrast, D425 cell proliferation relied much more heavily on *MYC* expression, and its disruption led to a rapid decrease in cell viability, underscoring strong *MYC* dependency.

A CRISPRi screen of the D425 ecDNA identified a putative *MYC* enhancer 436 kb 5’ of the *MYC* promoter, designated *ecMYC E1*, that displayed canonical enhancer hallmarks, including open chromatin, DNA looping to the *MYC* promoter, and H3K27ac histone mark occupancy in multiple *MYC*-amplified ecDNA- or HSR-positive G3-MB cell lines and tumor organoids. The prevalence of *ecMYC E1* and *MYC* promoter coamplification was not only restricted to *MYC*-amplified pediatric and adult tumor lines but also preferentially occurred in G3-MB tumors in which *MYC* expression was regulated on ecDNA or HSRs. Although several non–G3-MB tumors exhibited coamplification of these two elements, none displayed any active enhancer marks at *ecMYC E1*. Analysis of previously published ATAC-seq data from the developing fetal cerebellum failed to reveal *MYC* activation via *ecMYC E1* (48), suggesting that this interaction forms during *MYC*-amplified G3-MB tumor development, but not in other tumor types in which *MYC* is amplified.

Reconstruction efforts for the *MYC*-amplified G3-MB models highlighted the amplification of several known cell-type–specific *MYC* enhancers, many of which are in the ∼500 kb region 5’ to the *MYC* promoter (43). Any of these enhancers could be rewired to activate *MYC*, but for all G3-MB cases, it was the *ecMYC E1* enhancer that drove *MYC* transcription. *ecMYC E1* is a small ∼2 kb element within the 202 kb *CASC8* region (60). Only one of the four reconstructed ecDNAs fully amplified the *CASC8* region. All three others failed to amplify any part of the *CASC8* locus directly upstream of the *ecMYC E1* enhancer, and the fragment breakpoint that truncates *CASC8* was conserved to within 100 bp in all three models. In addition, three of the *MYC*-amplified G3-MB lines harbored coamplification of *OTX2* on chromosome 14 with *MYC* on chromosome 8.

The ecDNAs and HSRs sequenced from G3-MB exhibited complex rearrangements that incorporated segments of chromosomes 14 and 3 flanking the amplified *MYC* sequences derived from 8q24. Chromosomes 14 and 3 have been identified as fusion partners in recurrent translocations targeting *MYC* in other tumor types, suggesting that the inclusion of these segments into ecDNAs or HSRs was not a random event. Canonical t(8, 14) translocation events occur in Burkitt lymphoma, in which the immunoglobulin heavy chain enhancer is fused to exon 2 of the *MYC* coding sequence (61). However, the segments of chromosome 14 amplified in G3-MB ecDNAs did not contain regions associated with the immunoglobulin heavy chain enhancer, implying that these elements are not driving *MYC* expression in these cells. In turn, recurrent t(3, 8)(q26;q24) translocations occur in myelodysplastic syndrome and acute myeloid leukemia (62), relying on regulatory elements derived from *EVI1/MECOM* genes from chromosome 3 to upregulate *MYC* transcription. This *EVI1/MECOM* region is not amplified in G3-MB ecDNA or HSRs; instead, a ∼1 kb intronic portion of the chromosome 3 *CD96* gene was amplified. Notably, this region corresponds to a transposable LINE element, designated EIE14 (54). This element is amplified in the COLO320DM cell line and was shown to act as an enhancer of *MYC* transcription (54). However, investigation of EIE14 sequences in G3-MB cell lines revealed no hallmarks of an active enhancer, consistent with our findings that *ecMYC E1* is the main driver of *MYC* transcription in these models. These findings highlight the dominant roles of various enhancers across different tumor types. The reason for the preferential incorporation of segments from chromosomes 14 and 3 into the ecDNAs detected in G3-MBs remains unclear. This may reflect the spatial proximity of the participating chromosomes within the nucleus or regions of chromosomal microhomology, including transposable elements, that also predispose to translocations and chromosomal fusions.

Suppression of the *ecMYC E1* enhancer locus in multiple *MYC*-amplified G3-MB cell line and tumor organoid models led to a significant reduction in *MYC* transcription. However, silencing of the ec*MYC E1* locus was compensated for after prolonged cell culture by an increase in ecDNA copy number. This occurred only in DM- and not HSR-containing tumors, highlighting the ability of randomly segregating episomal elements to rapidly adapt to perturbations. The same phenomenon was also observed in tumors that amplify *DHFR* on ecDNA in response to methotrexate treatment (55) and in *FGFR2*-amplified tumors, in which ecDNA copy number increased as a compensatory response to FGFR2 inhibition (10). A recent article reported that after single-cell sorting for high or low ecDNA, each population returned to the same ecDNA copy number after only a few divisions (bioRxiv 2025.04.26.650733), but the mechanisms underlying cells’ selection for this specific copy number are still being investigated.

Activation of *MYC* transcription involved the binding of the basic helix–loop–helix transcription factor NeuroD1, together with the established *MYC* coactivator BRD4 (56, 57), to the *MYC* promoter and the *ecMYC E1* enhancer. Reciprocal Co-IP indicated a direct interaction between the two proteins, forming a regulatory complex. Recruitment of BRD4 by NeuroD1 to activate transcription was previously reported in small cell lung cancer (63), a neuroendocrine tumor, but not in other tumors, including medulloblastoma. Acute loss of either NeuroD1 or BRD4 activity led to a significant decrease in *MYC* transcription, and silencing of *ecMYC E1* displaced NeuroD1 binding, supporting the roles of both factors in G3-MB.

NeuroD1 was first identified as a factor that reprogrammed *Xenopus* ectoderm to form neurons (64), and it functions as a transcriptional and epigenetic regulator within mouse and human lineages, promoting a neuronal cell fate (58). As a pioneer transcription factor, NeuroD1 can bind heterochromatin, triggering the loss of repressive histone H3K27me3 marks and a concomitant gain of activating H3K27ac marks on proneural target genes. Loss of NeuroD1 impairs the differentiation of cerebellar granule neuronal progenitors from which medulloblastomas arise (65). As a member of the Atonal gene family, NeuroD1 can act combinatorially with a plethora of other transcription factors, including ATOH1/MATH1, which is similarly essential for granule neuron histogenesis (66). Although NeuroD1 can promote the differentiation of SHH-MB (67), its role in G3-MB and *MYC* regulation has not been previously documented.

Although BRD4 was found to be a potent therapeutic target in many *MYC*-overexpressing tumors (57), NeuroD1 has not yet been fully investigated as a therapeutic target in these tumors. Directly targeting transcription factors is particularly challenging and may require, for example, the development of molecular glues to limit their activity. Previous studies have suggested that ecDNAs need to be targeted specifically in combination with cytotoxic chemotherapies to reduce resistance or therapeutic escape by ecDNA-containing tumors (10). However, not all ecDNAs amplify therapeutically actionable targets, including the *MYC* family of proto-oncogenes. A possible mechanism for targeting ecDNA and ec*MYC E1* specifically in G3-MB would be to leverage the sequence-specific DNA-intercalating polyamide platform synthetic gene regulator developed at St. Jude (68). Targeting the enhancer would provide greater therapeutic benefit, as it would be tumor-cell specific and does not seem active in normal cells, thereby reducing the detrimental effects on normal development often observed with systemic therapy. Cells with increased ecDNA copy number are more sensitive to CHK1 inhibition than those without ecDNA, suggesting that a combination of CHK1 inhibition and direct targeting of the ecDNA might increase therapeutic responses (10). We found that targeting CDK4/6 and MYC acts synergistically to suppress G3-MB proliferation (69), further reinforcing the need to directly target ecDNA using a drug combination. Indeed, it is unlikely that targeting ecDNA or MYC alone as monotherapy would be efficient.

Chapman and collaborators investigated cell-type–specific *MYC* enhancers through a comparative study between the D458 and D283 G3-MB cell lines using a CRISPRi screen targeting 645 accessible loci (5). Our study leveraged the power of a CRISPRi screen by utilizing a library of more than 10,000 sgRNAs that saturate open chromatin regions defined by pan-tissue H3K27ac ChIP-seq from the ENCODE database (70), spanning the entire ∼3 Mb topologically associated domain containing the *MYC* locus. This enabled comprehensive interrogation of the *MYC* regulatory locus in an unbiased manner. Coupled with genomic architecture analyses, we identified a 2 kb *MYC* enhancer, termed *ecMYC E1*, in the same genomic neighborhood as the superenhancer reported by Chapman and colleagues (5). Chromatin accessibility analyses in many additional G3-MB cell lines and patient-derived tumor organoid models displayed conserved enhancer marks for this 2 kb *MYC* enhancer, supporting its specificity to this highly aggressive *MYC*-driven medulloblastoma subgroup. We did not detect open chromatin or activating histone marks at the *ecMYC E1* enhancer in any other tumor models, including those with *MYC* amplification or overexpression. Targeted functional validation of this enhancer region led to a loss of *MYC* transcription in all tested models, confirming its role in activating *MYC* in G3-MB. Mechanistically, we showed that NeuroD1 and BRD4 occupancy at *ecMYC E1* was required for its function, providing a model for how the *ecMYC E1* enhancer contacts the *MYC* promoter to facilitate *MYC* transcription.

In conclusion, our work has identified a conserved enhancer in *MYC*-amplified G3-MB tumors as a potential therapeutic target for suppressing MYC, which is considered “undruggable” (71), in a tumor-specific manner. We and others have highlighted the challenges in treating ecDNA-containing tumors with targeted, single-agent approaches, making the development and preclinical testing of combinatorial approaches to target ecDNAs warranted to prevent resistance by increasing ecDNA copy number.

## Supplementary Material

Supplementary Figure S1 Dependency Map results for MYC and OTX2 in the G3-MB D425 and D458 cell lines.

Supplementary Figure S2 AmpliconArchitect reconstructions of MYC-amplified G3-MB cell models.

Supplementary Figure S3 Genome-wide Hi-C sequencing displays ecMYC E1 looping to the MYC promoter.

Supplementary Figure S4 MYC amplification method by FISH imaging.

Supplementary Figure S5 H3K4me1 ChIP-seq in mouse ESCs and NPCs at the MYC locus.

Supplementary Figure S6 PVT1 promoter and MYC transcription in ecDNA-amplified G3-MB.

Supplementary Figure S7 Enhancer characterization of EIE14 in G3-MB.

Supplementary Figure S8 AmpliconArchitect reconstructions of D425 ecDNA after ecMYC E1 silencing.

Supplementary Figure S9 BRD4 binds to the MYC promoter in MYC-amplified G3-MB.

Supplementary Figure S10 NeuroD1 in G3-MB.

Supplementary Figure S11 NeuroD1 knockdown by siRNA.

Supplementary Table S1 List of primary and secondary antibodies.

Supplementary Table S2 List of sgRNA sequences for validation experiments.

Supplementary Table S3 List of sgRNA sequences for the CRISPR inhibition screen of D425.

Supplementary Table S4 Post-MAGeCK analysis CRISPRi screen results.

Supplementary Table S5 List of DNA qPCR primers.

## Data Availability

All St. Jude sample data are available upon request using the St. Jude web portal (https://pbtp.stjude.cloud), an interactive platform that allows the scientific community to explore datasets and request samples for their investigations. All sequencing data from PedDep samples can be accessed through the Gene Expression Omnibus (RRID: SCR_005012) database super series: GSE317671. All patient-derived sequencing samples can be accessed through the European Genome-phenome Archive: EGAS50000001608, EGAS50000001609, and EGAS50000001610. All other raw data are available upon request to the corresponding author (martine.roussel@stjude.org).
