# Supplementary material for: A Conserved Enhancer Locus in Extrachromosomal DNA and Homogeneously Staining Regions Activates MYC Transcription in Group 3 Medulloblastoma
Source: Cancer Res. 2026 Apr 22;86(13):3160–78. doi: 10.1158/0008-5472.CAN-25-4691 (PMC13202998; doi:10.1158/0008-5472.CAN-25-4691)
Supplement: Supplementary Figure S1 — Dependency Map results for MYC and OTX2 in the G3-MB D425 and D458 cell lines. [file can-25-4691_supplementary_figure_s1_suppsf1.pdf]

A

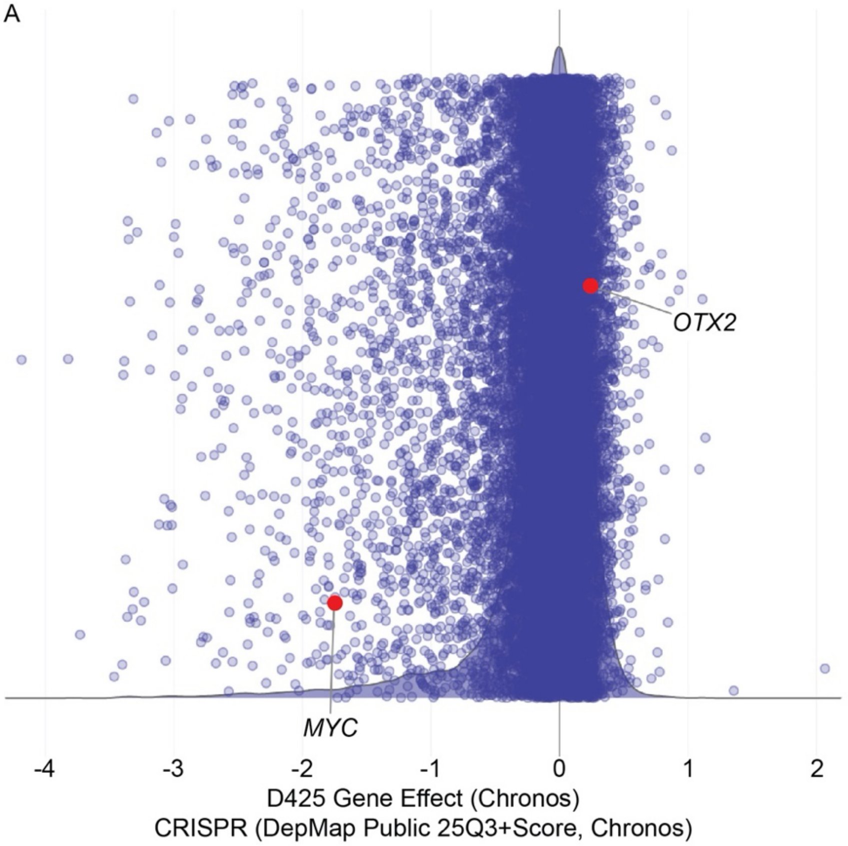

B

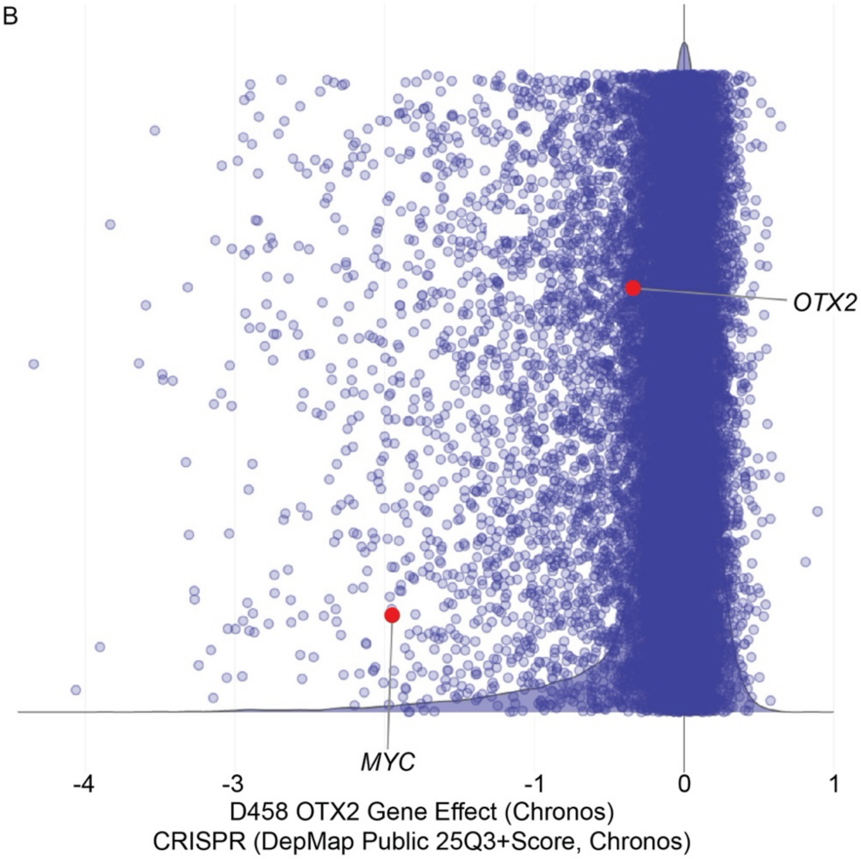

Supplementary Figure S1: Dependency Map results for *MYC* and *OTX2* in the G3-MB D425 and D458 cell lines

Results from the genome-wide CRISPR knockout screen of the (**A**) D425 and (**B**) D458 cell lines performed by the Cancer Dependency Map.
