# Supplementary material for: A Conserved Enhancer Locus in Extrachromosomal DNA and Homogeneously Staining Regions Activates MYC Transcription in Group 3 Medulloblastoma
Source: Cancer Res. 2026 Apr 22;86(13):3160–78. doi: 10.1158/0008-5472.CAN-25-4691 (PMC13202998; doi:10.1158/0008-5472.CAN-25-4691)
Supplement: Supplementary Figure S2 — AmpliconArchitect reconstructions of MYC-amplified G3-MB cell models. [file can-25-4691_supplementary_figure_s2_suppsf2.pdf]

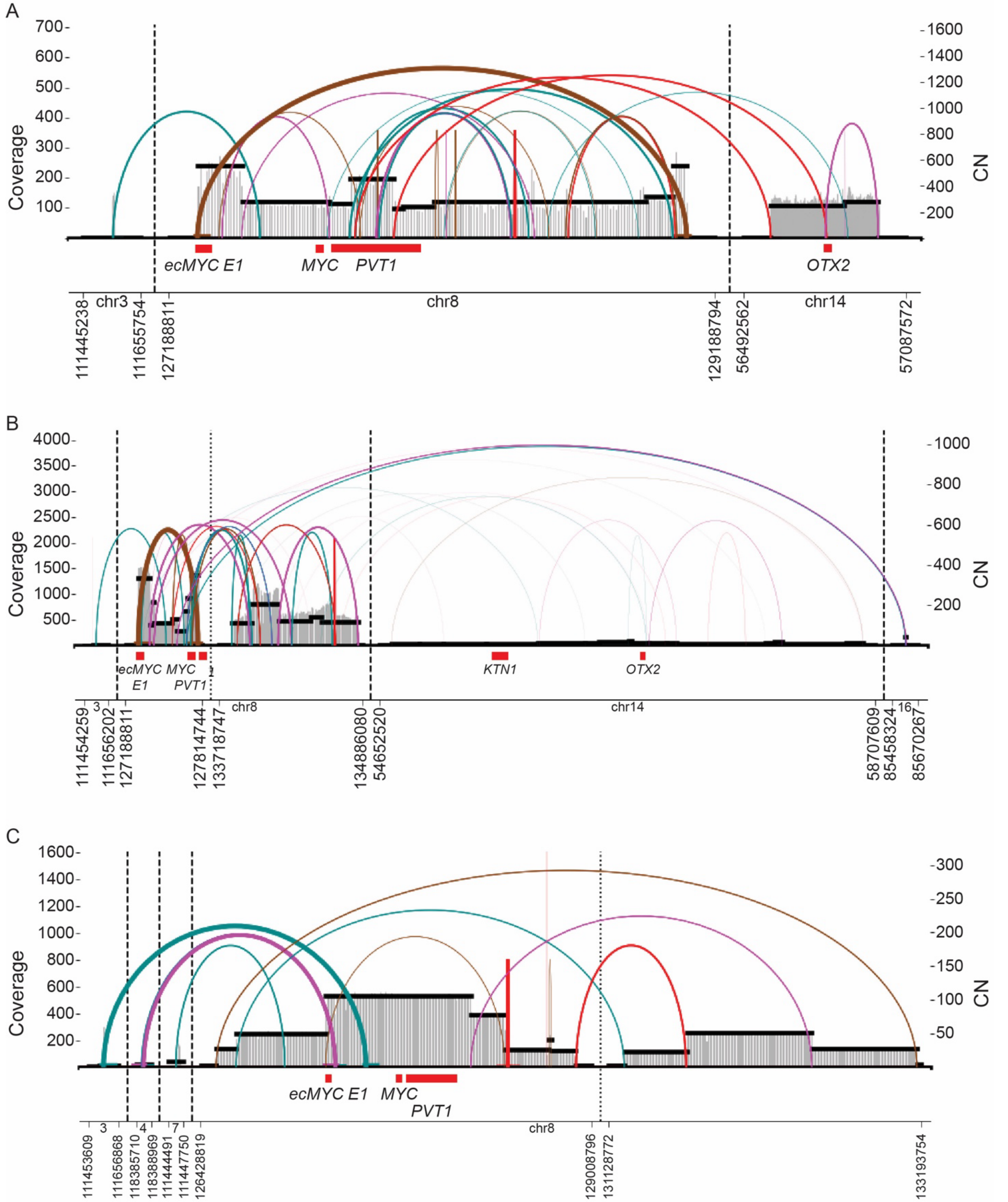

Supplementary Figure S2: AmpliconArchitect reconstructions of *MYC*-amplified G3-MB cell models
AmpliconArchitect reconstructions of *MYC*-amplified G3-MB models (**A**) D458, (**B**) HDMB03, and (**C**) SJMB016880.
