# Supplementary material for: A Conserved Enhancer Locus in Extrachromosomal DNA and Homogeneously Staining Regions Activates MYC Transcription in Group 3 Medulloblastoma
Source: Cancer Res. 2026 Apr 22;86(13):3160–78. doi: 10.1158/0008-5472.CAN-25-4691 (PMC13202998; doi:10.1158/0008-5472.CAN-25-4691)
Supplement: Supplementary Figure S3 — Genome-wide Hi-C sequencing displays ecMYC E1 looping to the MYC promoter. [file can-25-4691_supplementary_figure_s3_suppsf3.pdf]

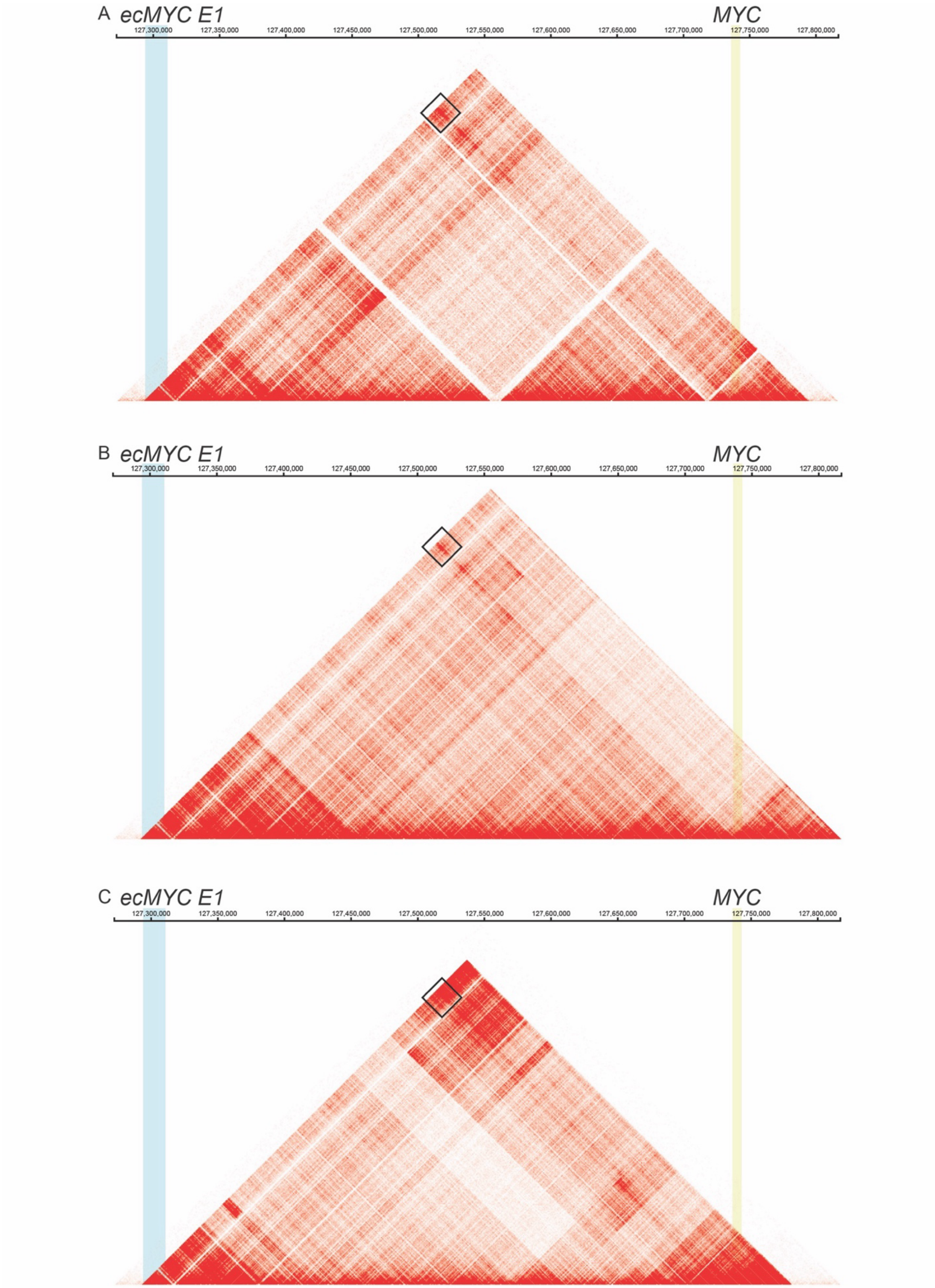

Supplementary Figure S3: Genome-wide Hi-C sequencing displays *ecMYC E1* looping to the *MYC* promoter

Genome-wide Hi-C for the (**A**) D425 and (**B**) D458 cell lines and (**C**) SJMB016880 tumor organoid. The black box identified the looping interaction between the *ecMYC E1* enhancer and the *MYC* promoter.
