# Supplementary material for: A Conserved Enhancer Locus in Extrachromosomal DNA and Homogeneously Staining Regions Activates MYC Transcription in Group 3 Medulloblastoma
Source: Cancer Res. 2026 Apr 22;86(13):3160–78. doi: 10.1158/0008-5472.CAN-25-4691 (PMC13202998; doi:10.1158/0008-5472.CAN-25-4691)
Supplement: Supplementary Figure S4 — MYC amplification method by FISH imaging. [file can-25-4691_supplementary_figure_s4_suppsf4.pdf]

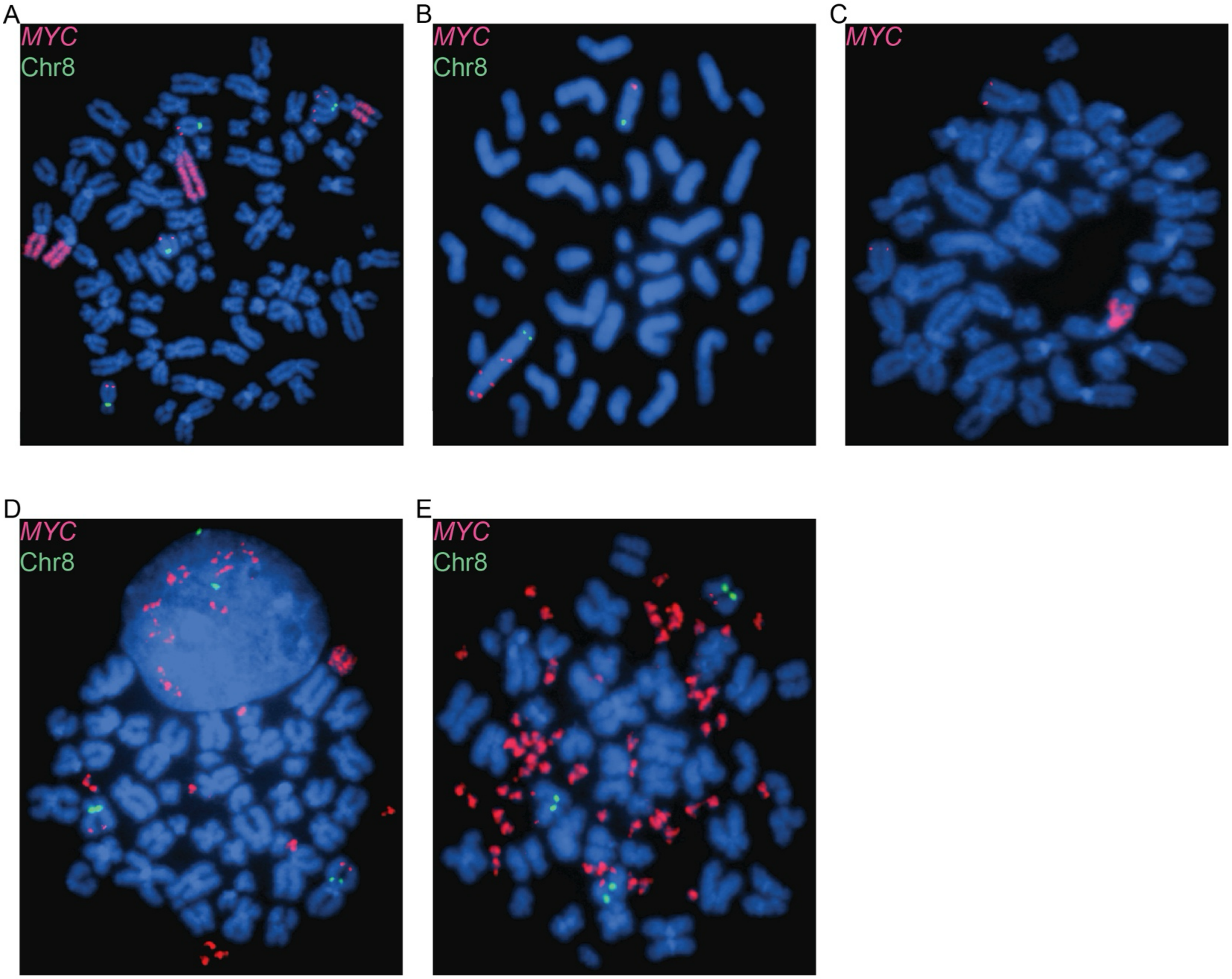

Supplementary Figure S4: *MYC* amplification method by FISH imaging
Fluorescence *in situ* hybridization imaging for *MYC* amplification in the G3-MB cell lines (**A**) HDMB03, (**B**) D283, (**C**) D341, and tumor organoids (**D**) SJMB016880, or (**E**) SJMB030315.
