# Supplementary material for: A Conserved Enhancer Locus in Extrachromosomal DNA and Homogeneously Staining Regions Activates MYC Transcription in Group 3 Medulloblastoma
Source: Cancer Res. 2026 Apr 22;86(13):3160–78. doi: 10.1158/0008-5472.CAN-25-4691 (PMC13202998; doi:10.1158/0008-5472.CAN-25-4691)
Supplement: Supplementary Figure S5 — H3K4me1 ChIP-seq in mouse ESCs and NPCs at the MYC locus. [file can-25-4691_supplementary_figure_s5_suppsf5.pdf]

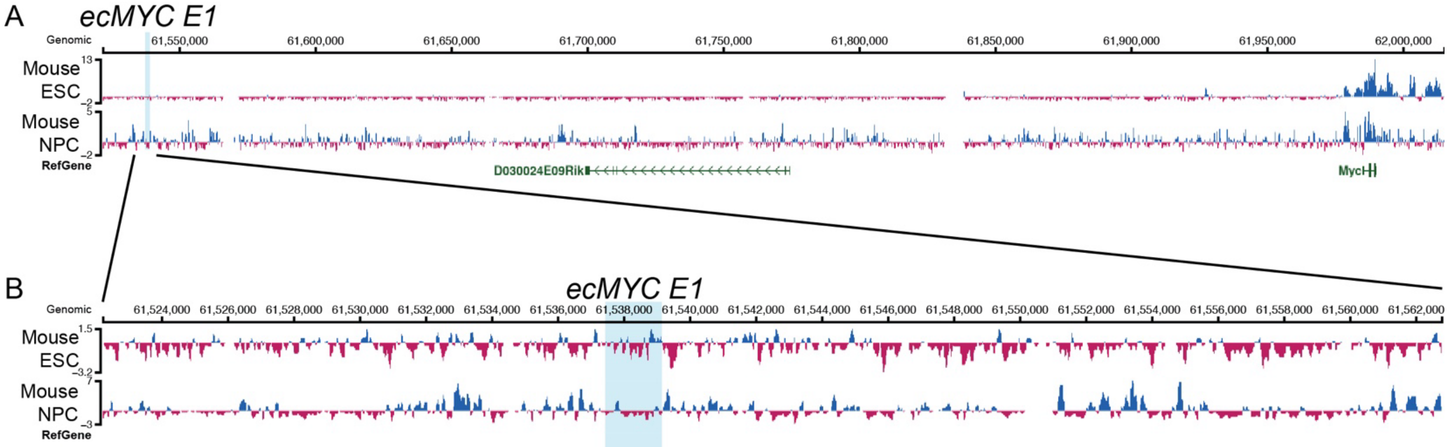

Supplementary Figure S5: H3K4me1 ChIP-seq in mouse ESCs and NPCs at the *MYC* locus

ChIP-seq coverage tracks for H3K4me1 in mouse epithelial stem cells (ESCs) (top track) and mouse neural progenitor cells (NPCs) (lower track) for (**A**) the *MYC* locus or (**B**) zoomed in to the *ecMYC E1* locus. The *ecMYC E1* locus is highlighted in blue in both panels.
