# Supplementary material for: A Conserved Enhancer Locus in Extrachromosomal DNA and Homogeneously Staining Regions Activates MYC Transcription in Group 3 Medulloblastoma
Source: Cancer Res. 2026 Apr 22;86(13):3160–78. doi: 10.1158/0008-5472.CAN-25-4691 (PMC13202998; doi:10.1158/0008-5472.CAN-25-4691)
Supplement: Supplementary Figure S6 — PVT1 promoter and MYC transcription in ecDNA-amplified G3-MB. [file can-25-4691_supplementary_figure_s6_suppsf6.pdf]

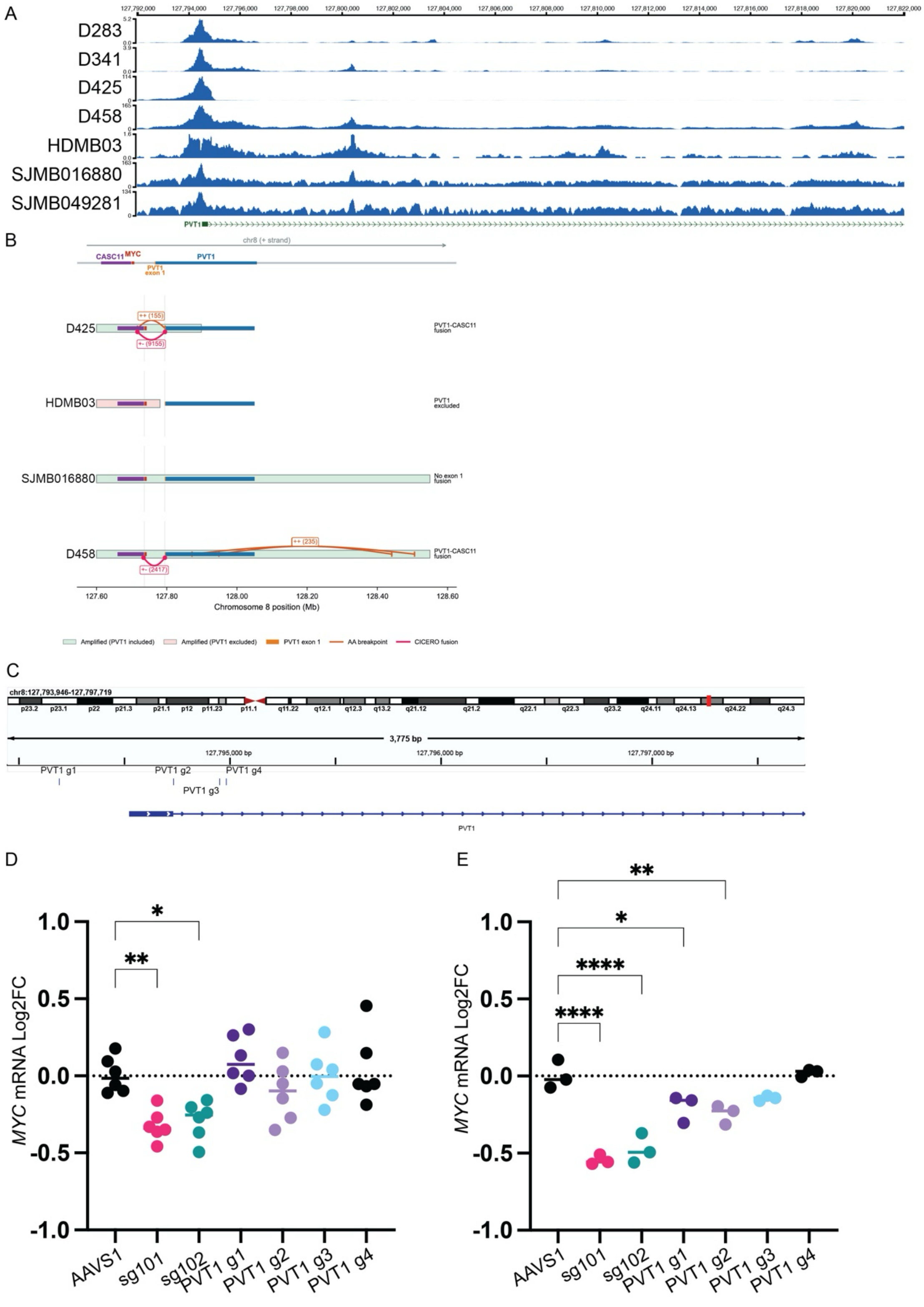

Supplementary Figure S6: *PVT1* promoter and *MYC* transcription in ecDNA-amplified G3-MB
(**A**) Aligned ATAC-seq tracks for the *PVT1* locus in *MYC*-amplified G3-MB: D283, D341, D425, D458, HDMB03 cell lines and SJMB016880, and SJMB049281 tumor organoids. (**B**) AmpliconArchitect reconstruction diagram for the *MYC-PVT1* locus for all tested G3-MB models. (**C**) Schematic showing the locations for the four *PVT1* promoter sgRNAs. RT-qPCR for *MYC* mRNA transcription at DT7 after targeted silencing of the *ecMYC E1* enhancer or *PVT1* promoter in the (**D**) D458 cell line or (**E**) SJMB016880 tumor organoid.
