# Supplementary material for: A Conserved Enhancer Locus in Extrachromosomal DNA and Homogeneously Staining Regions Activates MYC Transcription in Group 3 Medulloblastoma
Source: Cancer Res. 2026 Apr 22;86(13):3160–78. doi: 10.1158/0008-5472.CAN-25-4691 (PMC13202998; doi:10.1158/0008-5472.CAN-25-4691)
Supplement: Supplementary Figure S7 — Enhancer characterization of EIE14 in G3-MB. [file can-25-4691_supplementary_figure_s7_suppsf7.pdf]

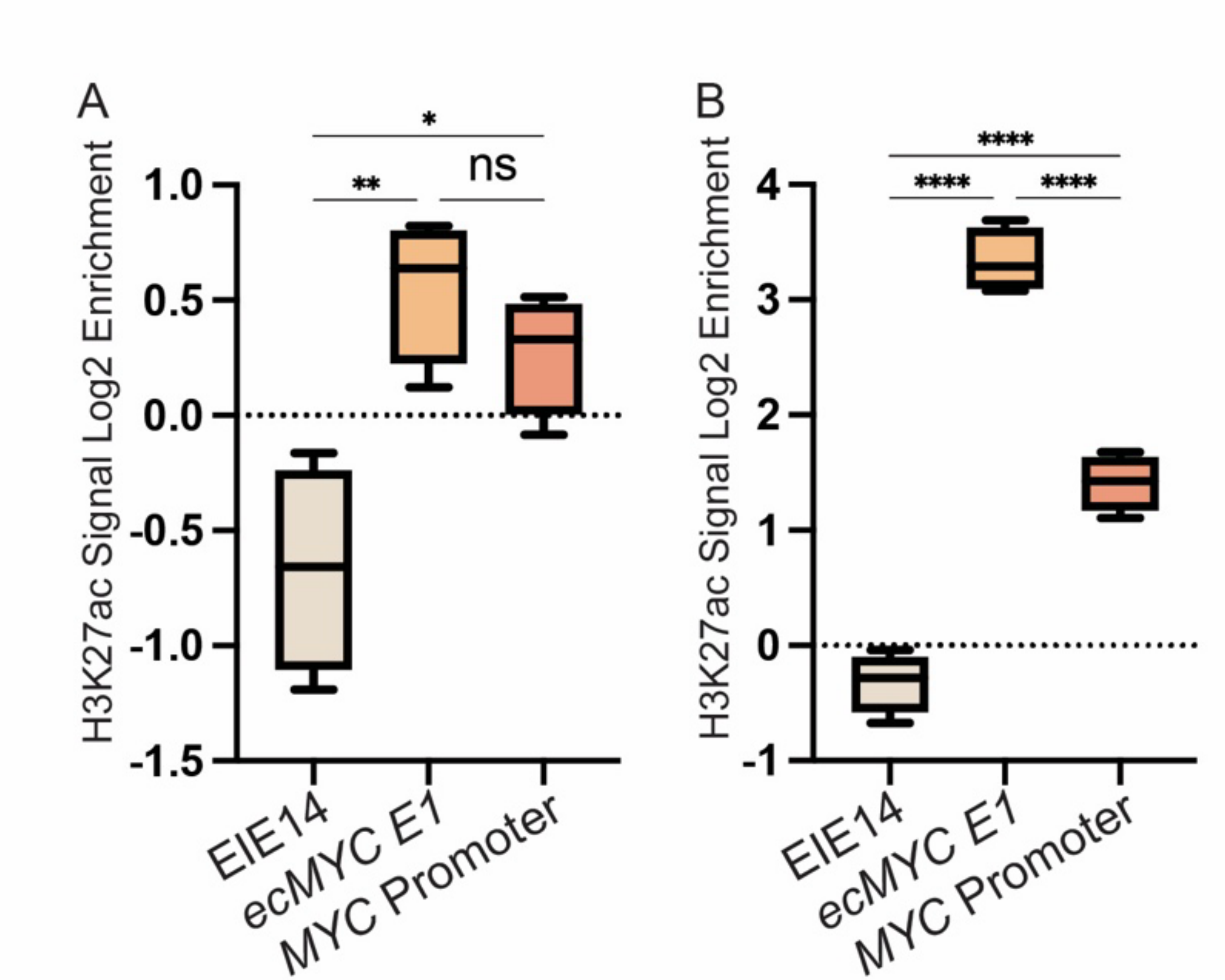

Supplementary Figure S7: Enhancer characterization of EIE14 in G3-MB
H3K27ac histone mark enrichment in the (**A**) D425 and (**B**) HDMB03 *MYC*-amplified G3-MB cell lines for EIE14, *ecMYC E1*, and the *MYC* promoter.
