# Supplementary material for: A Conserved Enhancer Locus in Extrachromosomal DNA and Homogeneously Staining Regions Activates MYC Transcription in Group 3 Medulloblastoma
Source: Cancer Res. 2026 Apr 22;86(13):3160–78. doi: 10.1158/0008-5472.CAN-25-4691 (PMC13202998; doi:10.1158/0008-5472.CAN-25-4691)
Supplement: Supplementary Figure S8 — AmpliconArchitect reconstructions of D425 ecDNA after ecMYC E1 silencing. [file can-25-4691_supplementary_figure_s8_suppsf8.pdf]

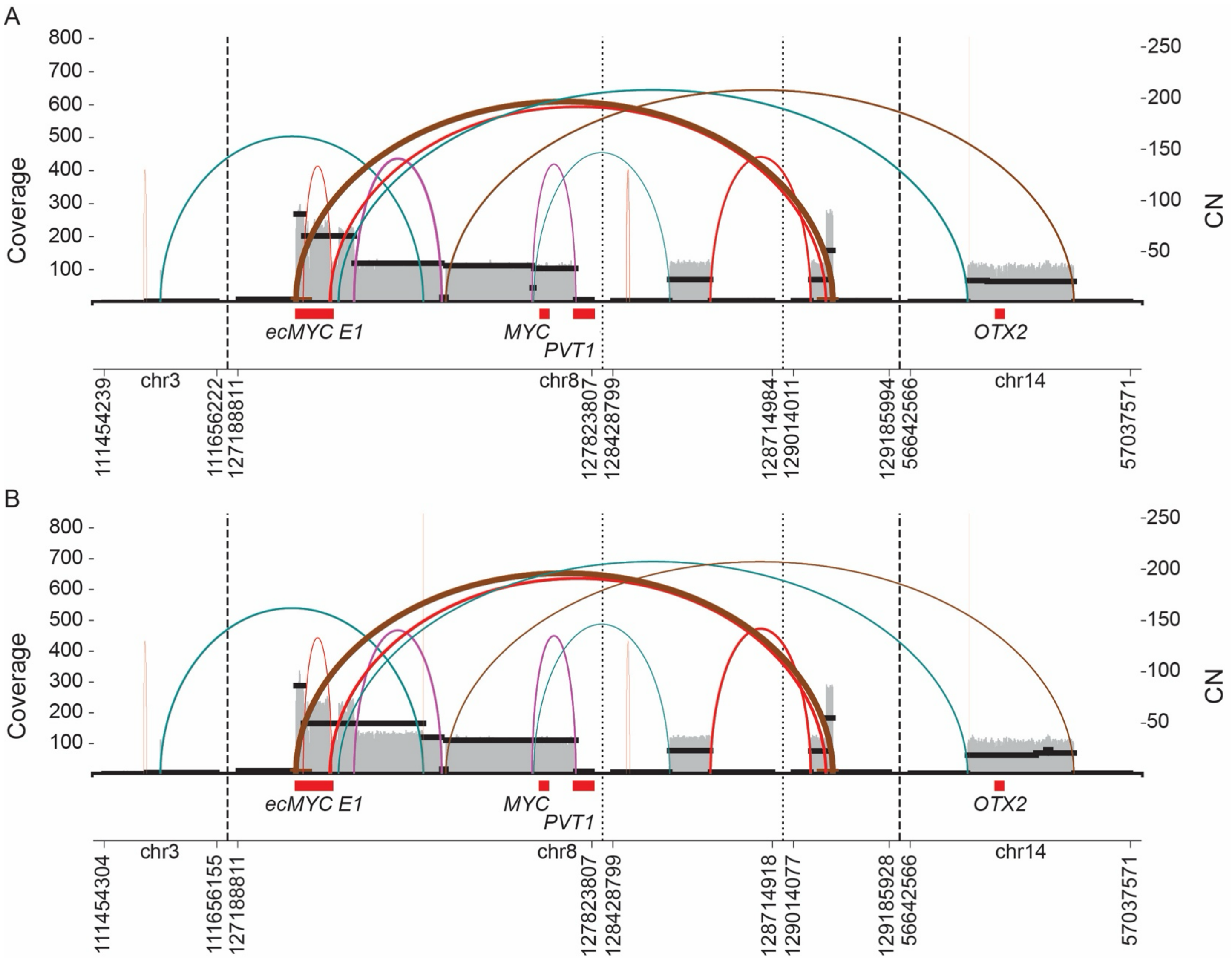

Supplementary Figure S8: AmpliconArchitect reconstructions of D425 ecDNA after *ecMYC E1* silencing

AmpliconArchitect reconstruction of the D425 ecDNA at doubling time 13 after silencing targeting (**A**) *AAVS1* or (**B**) *ecMYC E1*. CN = inferred copy number; coverage = depth of sequencing
