# Supplementary material for: A Conserved Enhancer Locus in Extrachromosomal DNA and Homogeneously Staining Regions Activates MYC Transcription in Group 3 Medulloblastoma
Source: Cancer Res. 2026 Apr 22;86(13):3160–78. doi: 10.1158/0008-5472.CAN-25-4691 (PMC13202998; doi:10.1158/0008-5472.CAN-25-4691)
Supplement: Supplementary Figure S9 — BRD4 binds to the MYC promoter in MYC-amplified G3-MB. [file can-25-4691_supplementary_figure_s9_suppsf9.pdf]

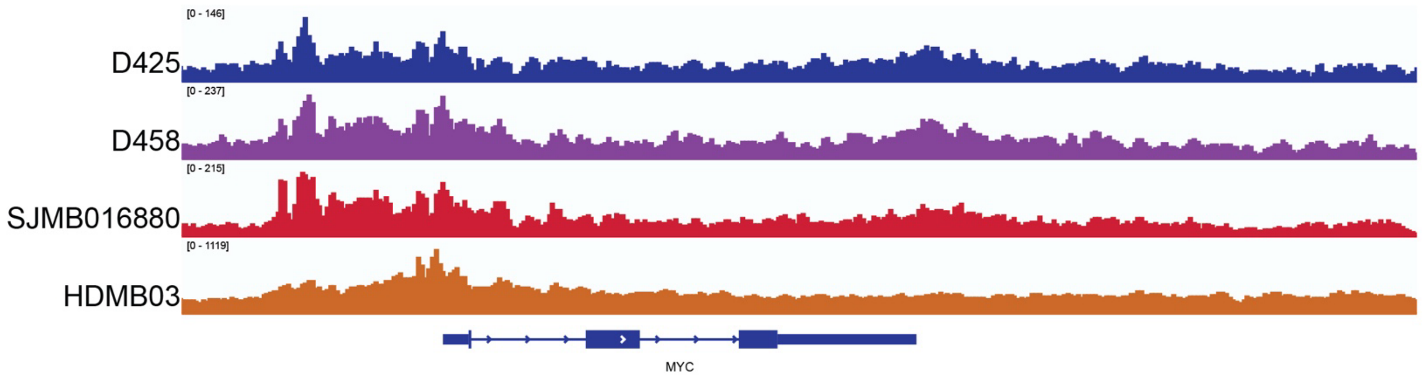

Supplementary Figure S9: BRD4 binds to the *MYC* promoter in *MYC*-amplified G3-MB
CUT&RUN targeting BRD4 at the *MYC* locus in the G3-MB cell lines D425 (blue), D458 (purple), HDMB03 (orange), and tumor organoid SJMB016880 (red).
