# Supplementary material for: A Conserved Enhancer Locus in Extrachromosomal DNA and Homogeneously Staining Regions Activates MYC Transcription in Group 3 Medulloblastoma
Source: Cancer Res. 2026 Apr 22;86(13):3160–78. doi: 10.1158/0008-5472.CAN-25-4691 (PMC13202998; doi:10.1158/0008-5472.CAN-25-4691)
Supplement: Supplementary Figure S10 — NeuroD1 in G3-MB. [file can-25-4691_supplementary_figure_s10_suppsf10.pdf]

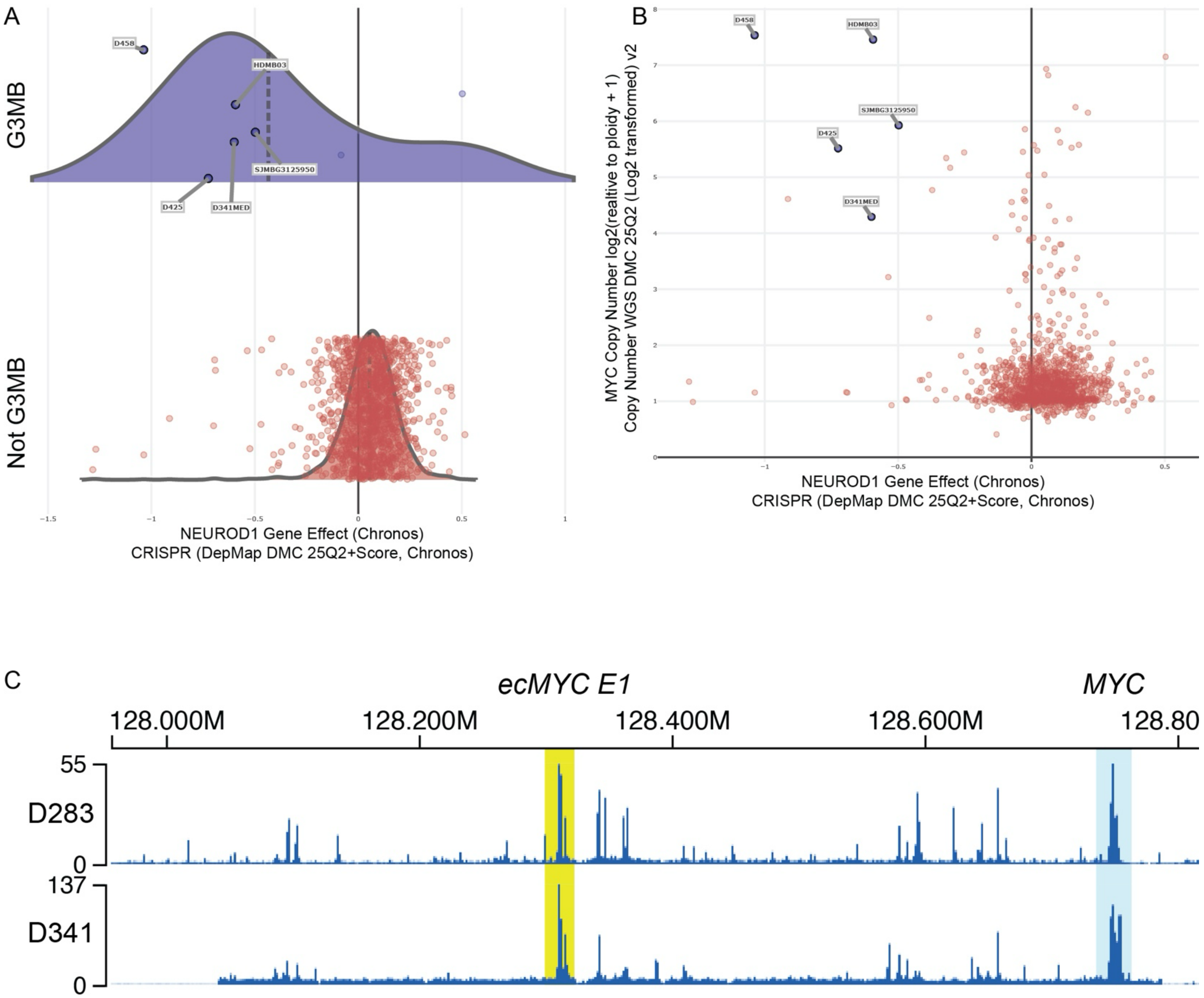

Supplementary Figure S10: NeuroD1 in G3-MB

(**A**) Results for NeuroD1 gene effect (x-axis) by DepMap CRISPR knockout screen of over 1000 cancer cell lines and cell models sorted by G3-MB (top, purple) and non-G3-MB (bottom, orange/pink). (**B**) Results for NeuroD1 gene effect (x-axis) by DepMap CRISPR knockout screen of over 1000 cancer cell lines and cell models sorted by *MYC* copy number (y-axis). G3-MB cell lines and cell models are annotated. (**C**) Publicly available ChIP-seq for NeuroD1 binding at the *ecMYC E1* and *MYC* loci in D283 (top track) and D341 (bottom track) G3-MB cell lines.
