# Supplementary material for: A Conserved Enhancer Locus in Extrachromosomal DNA and Homogeneously Staining Regions Activates MYC Transcription in Group 3 Medulloblastoma
Source: Cancer Res. 2026 Apr 22;86(13):3160–78. doi: 10.1158/0008-5472.CAN-25-4691 (PMC13202998; doi:10.1158/0008-5472.CAN-25-4691)
Supplement: Supplementary Figure S11 — NeuroD1 knockdown by siRNA. [file can-25-4691_supplementary_figure_s11_suppsf11.pdf]

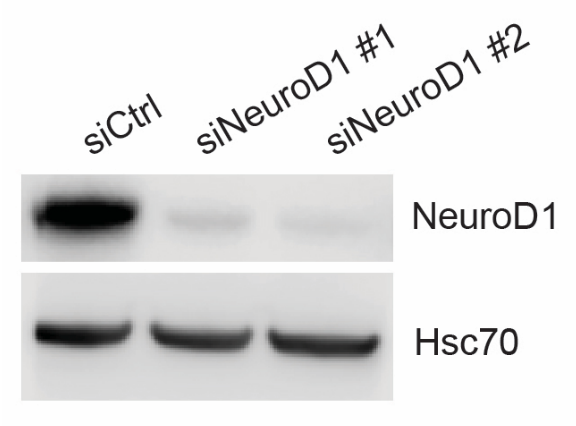

Supplementary Figure S11: NeuroD1 knockdown by siRNA
Immunoblot for NeuroD1 and Hsc70 in the D458 cell line 48 hours after transfection with siRNA. siNeuroD1 #1 = Thermo #s9457; siNeuroD1 #2 = Thermo #s9458 (**see Materials and Methods**).
